# Colloidally Stable P(DMA-AGME)-Ale-Coated Gd(Tb)F_3_:Tb^3+^(Gd^3+^),Yb^3+^,Nd^3+^ Nanoparticles as a Multimodal Contrast Agent for Down- and Upconversion Luminescence, Magnetic Resonance Imaging, and Computed Tomography

**DOI:** 10.3390/nano11010230

**Published:** 2021-01-16

**Authors:** Oleksandr Shapoval, Viktoriia Oleksa, Miroslav Šlouf, Volodymyr Lobaz, Olga Trhlíková, Marcela Filipová, Olga Janoušková, Hana Engstová, Jan Pankrác, Adam Modrý, Vít Herynek, Petr Ježek, Luděk Šefc, Daniel Horák

**Affiliations:** 1 Institute of Macromolecular Chemistry, Czech Academy of Sciences, 162 06 Prague 6, Czech Republic; oleksa@imc.cas.cz (V.O.); slouf@imc.cas.cz (M.Š.); lobaz@imc.cas.cz (V.L.); trhlikova@imc.cas.cz (O.T.); filipova@imc.cas.cz (M.F.); janouskova324@gmail.com (O.J.); 2 Institute of Physiology, Czech Academy of Sciences, 142 20 Praha 4, Czech Republic; hana.engstova@fgu.cas.cz (H.E.); Petr.Jezek@fgu.cas.cz (P.J.); 3 Center for Advanced Preclinical Imaging (CAPI), First Faculty of Medicine, Charles University, 120 00 Prague 2, Czech Republic; jan.pankrac@lf1.cuni.cz (J.P.); adam.modry@lf1.cuni.cz (A.M.); vit.herynek@lf1.cuni.cz (V.H.)

**Keywords:** up-conversion luminescence, down-conversion luminescence, colloidal stability, nanoparticles, MRI, computed tomography

## Abstract

Multimodal imaging, integrating several modalities including down- and up-conversion luminescence, *T*_1_- and *T*_2_(*T*_2_*)-weighted MRI, and CT contrasting in one system, is very promising for improved diagnosis of severe medical disorders. To reach the goal, it is necessary to develop suitable nanoparticles that are highly colloidally stable in biologically relevant media. Here, hydrophilic poly(*N*,*N*-dimethylacrylamide-*N*-acryloylglycine methyl ester)-alendronate-[P(DMA-AGME)-Ale]-coated Gd(Tb)F_3_:Tb^3+^(Gd^3+^),Yb^3+^,Nd^3+^ nanoparticles were synthesized by a coprecipitation method in ethylene glycol (EG) followed by coating with the polymer. The particles were tho-roughly characterized by a dynamic light scattering (DLS), transmission electron microscopy (TEM), Fourier-transform infrared spectroscopy (FTIR), thermogravimetric analysis (TGA), X-ray energy dispersive spectroscopy (EDAX), selected area electron diffraction (SAED), elemental ana-lysis and fluorescence spectroscopy. Aqueous particle dispersions exhibited excellent colloidal stability in water and physiological buffers. In vitro toxicity assessments suggested no or only mild toxicity of the surface-engineered Gd(Tb)F_3_:Tb^3+^(Gd^3+^),Yb^3+^,Nd^3+^ particles in a wide range of concentrations. Internalization of the particles by several types of cells, including HeLa, HF, HepG2, and INS, was confirmed by a down- and up-conversion confocal microscopy. Newly developed particles thus proved to be an efficient contrast agent for fluorescence imaging, *T*_1_- and *T*_2_(*T*_2_*)-weighted magnetic resonance imaging (MRI), and computed tomography (CT).

## 1. Introduction

With the rapid development of science and technology, multimodal imaging is attracting increasing attention because it can integrate advantages of different imaging modes in one system and improve the efficiency of diagnosis and biomedical research [1]. Multimodal imaging can thus overcome limitations of single imaging platforms, such as spatial and temporal resolution, depth penetration or sensitivity. These limitations are inherent in routine clinical investigations, including magnetic resonance imaging (MRI), positron emission tomography, computed tomography (CT), optical fluorescence imaging (FI), and sonography. Multimodal imaging plays also an important role in recognition of the tumor border and may help effectively guide the surgical resection in clinical practice dealing with cardiovascular, neuropsychiatric, and other disorders [1,2,3].

In these medical applications, the nanoparticles are often used, which have advantage of a large surface area to be functionalized with multimodal reporters and targe-ting vectors. Examples of nanosized particles used in imaging technologies include dendrimers, liposomes, gold, silver, quantum dots, iron oxides, silica, as well as lanthanides [2,4]. Among the lanthanide-based platforms for fluorescence imaging (FI), a special attention is paid to fluorides, because they have lower vibrational energy than oxides, and consequently, the quenching of the exited state of Ln cations is minimized, which results in high quantum efficiency of the luminescence [5]. In recent years, a lot of research has been focused on application of down- (DC) and up-conversion (UC) lanthanide fluorides [3,4,6,7]. Compared to traditionally used fluorescent organic dyes and quantum dots, lanthanide-based fluorides have a number of advantages, such as sharp emission bandwidth, long lifetime, tunable emission, high photostability, low cytotoxicity, and low background autofluorescence for DC and UC fluorescence. They are also interesting as contrast agents for MRI and CT due to paramagnetic properties and X-ray contrast [8]. Combining fluorescence with magnetic and X-ray properties of lanthanides is attractive, because greater sensitivity and resolution of FI complement MRI and CT. Particularly, MRI is superior for imaging of soft tissues because of abundance of protons, whereas X-ray CT is a powerful tool for detection of hard body parts with high electron densities. Moreover, CT is available for patients with metal implants, whereas MRI not, and MRI does not use ionizing radiation and iodinated contrast agents, in contrast to X-ray CT. Therefore, combination of FI, MRI, and CT introduces great benefits both for clinical diagnosis and biomedical research. 

A lot of effort is required to develop multipurpose nanoparticles with several pro-perties combined in a single particle. To date, various nanosized contrast enhancing agents have been reported, such as Fe [9,10,11], Au [4,12], Mn, or lanthanides [2,13,14], which are suitable for dual or multimodal FI, MRI, and CT imaging; however, only lanthanides have been demonstrated to be promising for ultrahigh field MRI [15,16]. More-over, lanthanide-based X-ray contrast agents are less nephrotoxic compared to the clinically used iodinated compounds [17]. Among the lanthanide ions, Yb^3+^, Gd^3+^, and Tb^3+^ play a special role due to a short electronic relaxation time and large effective magnetic moment (*μ*_eff_ = 7.9–9.7 μB), which makes them promising as potential *T*_1_ or *T*_2_(*T*_2_*) MRI contrast agents [18]. In addition, they have a large atomic number and high K-edge energy (~50–60 keV), which renders large X-ray attenuation coefficient. This well matches with the X-ray spectra used in clinical CT, thus enabling both high intrinsic contrast and rather low radiation exposure to the patients. Moreover, Tb^3+^ ions have attracted a particular attention due to their highly efficient green photoluminescence and a long luminescence lifetime, which results in applications in biochemical imaging without any autofluorescence background [19]. As Tb^3+^ does not have energy levels, which can directly absorb NIR light, Nd^3+^ and Yb^3+^ is the best choice to obtain UC luminescence in Tb^3+^-doped systems [20]. Moreover, unique optical properties of Nd and Yb have been the focus of numerous investigations for application in semiconductors and magnetic devices [4,21]. Introduction of Nd^3+^ ions in the host lattice increases the saturation magnetization of the nanoparticles. Co-doping of fluoride host with Yb^3+^ and Gd^3+^ ions can efficiently change transverse relaxivity and optimize both longitudinal and transverse relaxivities for *T*_1_/*T*_2_ dual-weighted MRI [22]. Advantage of Gd^3+^ ions lies in their very low leaching from the particles in water [23]. Gd-, Tb-, Yb-, and Nd-based particles are more stable than other MRI contrast agents, such as chelates, providing a particularly useful platform for the design of multimodal imaging nanoprobes. 

Generally, Gd- and Tb-based fluoride nanoparticles can be prepared by thermal decomposition, coprecipitation, complex-assisted hydrothermal methods, or reverse micelle and microwave-assisted techniques [24]. Nevertheless, post-synthesis heat treatment and functionalization are required in some of these methods in order to make the particles water-dispersible; however, this often leads to formation of very toxic byproducts. Typical advantages of precipitation in glycol-based media (e.g., ethylene glycol—EG or glycerol) are the capping abilities of glycols, their low toxicity compared to other organic solvents, and simplicity of the procedure [25]. EG is also used to control the particle size and morphology, as well as to prevent particle aggregation [26]. Preventing of aggregation could offer precise regulation of the physicochemical properties of the nanomaterials in any bio-application, as well as in vivo safety. 

One of the main challenges for applications of nanoparticles in biological media is to control the surface chemistry by introduction of specific coatings that have to be nontoxic, biocompatible, and allow binding of drugs, proteins, enzymes, antibodies, or nucleotides. This is associated with other requirements: (i) stabilization of nanoparticles in biological media at a high salt concentration, (ii) introduction of functional groups on the surface for further modification, and finally (iii) prevention of immediate uptake by the macrophages. Functionalization of nanoparticles additionally changes their properties and affects bioactivity. Hence, the right choice of the coating material with defined physicochemical and biochemical properties is extremely important to render the nanoparticles applicable in nanomedicine. For this purpose, different strategies have been reported, including the use of stabilizers, such as poly(ethylene glycol), poly(*N*–isopropylacrylamide), polyvinylpyrrolidone, poly(vinyl alcohol), poly(4-styrenesulfonic acid-*co*-maleic acid), and poly(*N*,*N*-dimethylacrylamide) [14,27,28]. Polyacrylamides seem to be a smart choice as a nanoparticle coating, as they provide not only colloidal stability in biological media, but also thermoresponsive behavior. 

In this report, we present co-doping of fluoride contrast agents with Gd^3+^, Tb^3+^, Yb^3+^, and Nd^3+^ ions to provide DC/UC luminescence, MRI, and CT contrast. We have synthesized copolymers of *N*,*N*-dimethylacrylamide and *N*-acryloylglycine methyl ester (AGME) as a coating, which not only provided biocompatibility, but also long-term colloidal stability in physiological media, such as phosphate-buffered saline (PBS) or sodium acetate. In vitro cytotoxicity and intracellular uptake of the particles was investigated using primary and cancer cell lines by PrestoBlue™ reagent and confocal microscopy, respectively. Different modalities of in vivo imaging on model mice then proved applicability of the particles for multimodal contrasting, in particular *T*_1_- and *T*_2_*-weighted MRI, computed tomography, and fluorescence imaging. 

## 2. Materials and Methods 

### 2.1. Materials

**Figure 1:**
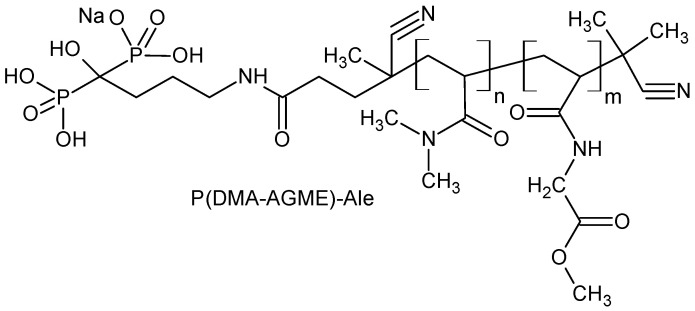
Chemical structure of alendronate-modified poly(*N*,*N*-dimethylacrylamide-*co-N*-acryloylglycine methyl ester).

Chloride hexahydrates of terbium(III), neodymium(III), ytterbium(III), and gadoli-nium(III) (99.99%), sodium tetrafluoroborate, phosphate-buffered saline (PBS), and *N*,*N*’-bis(2,3-dihydroxypropyl)-5-[*N*-(2,3-dihydroxypropyl)acetamido]-2,4,6-triiodoisophthal-amide (Iohexol) were obtained from Sigma-Aldrich (St. Louis, MO, USA). Ethanol (99%), ethylene glycol (EG), acetic and hydrochloric acids were obtained from Lach-Ner (Neratovice, Czech Republic). Sodium hydroxide and sodium chloride were purchased from Lachema (Brno, Czech Republic). Sodium acetate (NaAc) buffer (pH 5) was prepared from sodium hydroxide and acetic acid. Gibco™ penicillin-streptomycin, CellMask™ deep red and PrestoBlue™ cell viability reagents were obtained from Thermo Fisher Scientific (Waltham, MA, USA). Sulfo-Cyanine7 NHS ester (Cy7) was purchased from Lumiprobe (Hannover, Germany). Isoflurane was obtained from Baxter (San Juan, Puerto Rico). Alendronate-modified poly(*N*,*N*-dimethylacrylamide-*co-N*-acryloylglycine methyl ester) [P(DMA-AGME)-Ale; *M*_w_ = 11,000 g/mol] (Figure 1) was synthesized according to previously published procedure [29]. All chemicals were used directly without further purification. Ultrapure Q-water ultra-filtered on a Milli-Q Gradient A10 system (Millipore; Molsheim, France) was used in all experiments. 

### 2.2. Synthesis of Gd(Tb)F_3_:Tb^3+^(Gd^3+^),Yb^3+^,Nd^3+^ Nanoparticles 

Lanthanide fluoride nanoparticles were prepared by a coprecipitation method in EG. In a typical procedure, P(DMA-AGME)-Ale (10^−4^–10^−2^ mg/mL) was dissolved in EG (10 mL) with magnetic stirring to form a clear solution, to which 1 mmol of lanthanide chlorides (GdCl_3_·6H_2_O, TbCl_3_·6H_2_O, YbCl_3_·6H_2_O, and NdCl_3_·6H_2_O) was added. After vigorous stirring for 30 min, EG (3 mL) containing NaBF_4_ (3 mmol) was added dropwise and the mixture was kept at 75 °C (optionally 100 or 140 °C) for 3.5 h with stirring. The solution was cooled in air to room temperature (RT), the precipitate was separated by centrifugation (14,500 rpm; 30 min) and washed with water and ethanol several times. Finally, the product, denoted as Gd(Tb)F_3_:Tb^3+^(Gd^3+^),Yb^3+^,Nd^3+^@P(DMA-AGME)-Ale, was dispersed in 5 mL of water. One part of the dispersion was dried at 23 °C for 3 days under vacuum to obtain a sample for physicochemical analysis. Typical particles used in the experiments had molar composition GdF_3_:10%Tb^3+^,5%Yb^3+^,5%Nd^3+^ and TbF_3_:20%Gd^3+^,5%Yb^3+^,5%Nd^3+^. 

### 2.3. Modification of GdF_3_:Tb^3+^,Yb^3+^,Nd^3+^@P(DMA-AGME)-Ale Nanoparticles with Cy7-Ale 

Cy7-NHS (1 mg) was added to Ale (3.2 mg; tenfold molar excess) solution in PBS (1 mL) and the mixture was aged at RT for 24 h in the dark resulting in Cy7-Ale. The purified P(DMA-AGME)-Ale-coated GdF_3_:Tb^3+^,Yb^3+^,Nd^3+^ particles (30 mg/mL) were reacted with Cy7-Ale (l mg/mL) in PBS (pH 7.4) at RT overnight in the dark. Unbound Cy7 was removed by washing with PBS and water using centrifugation. 

### 2.4. Characterization of Nanoparticles

The particle morphology was examined on a Tecnai Spirit G2 transmission electron microscope (TEM; FEI; Brno, Czech Republic). Particle size distribution characterized by dispersity (*Ð*) was obtained by counting 300 particles from the TEM micrographs to determine the number-(*D_n_*) and weight-average particle diameter (*D_w_*) [29]:

*Ð* = *D*_w_/*D*_n_, (1)

*D_n_* = ∑N_i_*D*_i_/∑N_i_ (2)

*D_w_* = ∑N_i_*D*_i_^4^/∑N_i_*D*_i_^3^ (3)

where *D_i_* and N_i_ are the diameter and number of i-th particle, respectively. TEM microscope was equipped with an EDX energy dispersive spectrometer (EDAX; Mahwah, NJ, USA) used for analysis of the elemental composition of the nanoparticles. The crystal structure was verified by a selected area electron diffraction (SAED) as described in our previous work [30]. 

Dynamic light scattering (DLS) measurements (Zetasizer Ultra; Malvern, UK) provided the hydrodynamic diameter (*D*_h_) and polydispersity (*PD*) calculated from the cumulant analysis of the intensity autocorrelation, assuming a single particle size mode. Single exponential fit was applied to the autocorrelation function and the polydispersity was derived from the width of the assumed Gaussian distribution. Thermogravimetric analysis (TGA) of the particles was performed with a Perkin Elmer TGA 7 analyzer (Norwalk, CT, USA) over the temperature range 30–850 °C at a constant heating rate of 10 °C/min under nitrogen atmosphere. Content of Gd^3+^, Tb^3+^, Nd^3+^, and Yb^3+^ was determined using a SPECTRO XEPOS energy-dispersive X-ray fluorescence spectrometer (SPECTRO Analytical Instruments; Kleve, Germany) on samples digested with HNO_3_ in a Biotage Initiator microwave reactor (Biotage; Uppsala, Sweden). Emission and excitation spectra were recorded with a FS5 Edinburgh Instruments spectrofluorometer (Edinburgh, UK) coupled with UV and 980 nm CW laser with 2 W output power (MDL-III-980).

### 2.5. In Vitro Cytotoxicity 

Cytotoxicity of particles was tested using cancer and primary cell lines. Human cervical adenocarcinoma (HeLa cell line) and primary human dermal fibroblasts (HF cell line), kindly provided by Dr. Mělková and Dr. Dvořánková, respectively, the First Faculty of Medicine, Charles University, Prague, were cultivated in Dulbecco’s Modified Eagle Medium (DMEM) supplemented with 10% heat-inactivated fetal bovine serum (FBS) and 1% Gibco™ penicillin-streptomycin at 37 °C under 5% CO_2_ atmosphere. The cells (5 × 10^3^ of HeLa or 8 × 10^3^ of HF cells) were seeded onto 96-well flat-bottom plates (TPP; Prague, Czech Republic) in the medium (100 μL) for 24 h, aqueous dispersion of Gd(Tb)F_3_:Tb^3+^(Gd^3+^),Yb^3+^,Nd^3+^@P(DMA-AGME)-Ale nanoparticles (0.016–2 mg/mL) was added, cultivation continued for 72 h, and PrestoBlue™ (10 μL) was added for 3.5 h with HeLa or for 4 h with HF cells. The active component of the PrestoBlue^TM^ reagent (resa-zurin) was reduced to the highly fluorescent resorufin only in viable cells. Fluorescence was measured on a Synergy Neo plate reader (Bio-Tek; Prague, Czech Republic). As a control, cells in the absence of particles were used. For statistical analysis, GraphPad Prism software was used. The experiments were repeated two times in triplicates.

### 2.6. Downconversion Confocal Laser Scanning Microscopy 

In the investigation of uptake of the nanoparticles by HeLa and HF cells, they were seeded at a density of 150,000 cells per mL of culture medium on a glass dish with 4 chambers (20 mm microwell, cover glass with thickness 0.13–0.16 mm; Bio-Port Europe; Prague, Czech Republic) and cultivated at 37 °C for 24 h in 5% CO_2_ atmosphere. P(DMA-AGME)-Ale-coated Gd(Tb)F_3_:Tb^3+^(Gd^3+^),Yb^3+^,Nd^3+^ nanoparticles (1 mg/mL) were then added and incubated with the cells for 24 h, which was followed by washing with PBS and labeling with CellMask^TM^ deep red plasma membrane marker (1 µg/mL) at RT for 15 min. The cells were then washed with PBS twice and visualized using an FV10-ASV Olympus laser scanning confocal microscope equipped with a 60× oil immersion objective (Olympus; Prague, Czech Republic). The nanoparticles were detected at 405 nm excitation with 450–550 nm emission filter, while the plasma membrane was detected at 485 nm excitation with 520–600 nm emission filter.

### 2.7. Upconversion Confocal Laser Scanning Microscopy 

Human hepatocellular carcinoma HepG2 (ECACC 85011430) cells were cultivated in DMEM with 3 mM glutamine, 10% (*v*/*v*) fetal calf serum, 10 mM HEPES, 100 IU/mL peni-cillin, streptomycin (100 μg/mL), and 5 mM glucose at 37 °C in humidified air with 5% CO_2_. Rat insulinoma INS-1E cells (kindly provided by Prof. Maechler, University of Geneva, or purchased from AddexBio, cat. No. C0018009; San Diego, CA, USA) were cultured in 11 mM glucose and RPMI 1640 medium supplemented with 5% (*v/v*) fetal calf serum, 10 mM HEPES, 1 mM pyruvate, 50 µM mercaptoethanol, 50 IU/mL penicillin, and 50 mg/mL streptomycin. The cells were cultured on poly(L-lysine)-coated glass coverslips in DMEM (2 mL) for 2 days, incubated with GdF_3_:Tb^3+^,Yb^3+^,Nd^3+^@P(DMA-AGME)-Ale particle dispersion (200 μL; 10 mg/mL) for 24 h, transferred to a thermostable chamber at 37 °C under 5% CO_2_ atmosphere, and finally observed in a Leica TCS SP8 AOBS confocal inverted fluorescence microscope (Wetzlar, Germany) equipped with a HC PL APO 63×/1.20 NA W CORR CS2, WD = 0.3 mm, objective. For the measurement of upconversion luminescence, the particles were excited by a Chameleon Ultra I pulsed infrared tunable laser with wavelength range 690–1040 nm, maximum output power 4 W, pulse frequency 80 MHz, pulse width ~140 fs, and laser intensity controlled by electrooptical EOM modulator (Coherent; Santa Clara, CA, USA) and attenuator at 808 and 980 nm excitation. CellMask™ deep red-stained cell plasma membranes were visualized in a standard fluorescence confocal microscope with excitation and emission at 649 and 666 nm, respectively, using a WLL2 supercontinuous pulsed laser (NKT Photonics; Birkerød, Denmark) with an average laser power ~1.5 mW. 

### 2.8. In Vitro Longitudinal (T_1_) and Transversal Relaxation (T_2_) and Relaxivity (r_1,2_) Measurement 

Aqueous dispersions of Gd(Tb)F_3_:Tb^3+^(Gd^3+^),Yb^3+^,Nd^3+^@P(DMA-AGME)-Ale nanoparticles doped with different concentrations of Gd^3+^, Tb^3+^, Nd^3+^, and Yb^3+^ were subjected to MR relaxometry. *T*_1_ and *T*_2_ relaxation times responsible for contrast in MR images were measured on a 0.5 T Minispec relaxometer (Bruker BioSpin, Ettlingen, Germany). Relaxation times (*T*_1,2_) were converted to relaxivity rates (*R*_1,2_) and, after deducting the contribution of water, related to the actual lanthanide concentration *c*:

*R*_1,2_ = 1/*T*_1,2_ (4)

*r*_1,2_ = (1/*T*_1,2_ − 1/*T*_1,2water_)/*c* (5)

Relaxation times of GdCl_3_, TbCl_3_, NdCl_3_, and YbCl_3_ solutions were measured and respe-ctive relaxivities were calculated for comparison.

### 2.9. In Vivo Magnetic Resonance Imaging (MRI) 

The mice (CD1; two animals in each group; obtained from the Center for Experimental Biomodels, First Faculty of Medicine, Charles University, Prague, Czech Republic) were scanned both before and 0.5, 2, 5, 24, 96, and 168 h after retroorbital administration of the Gd(Tb)F_3_:Tb^3+^(Gd^3+^),Yb^3+^,Nd^3+^@P(DMA-AGME)-Ale nanoparticles (120 µL, 27 mg/mL of PBS). MR images were obtained using an MR imager Icon (Bruker BioSpin; Ettlingen, Germany) working at 1 T magnetic field. The animals were placed on a heated bed with a whole-body radiofrequency coil. Vital functions were monitored during the scanning and two sequences with different weighting were used to obtain coronal slices of the mouse body: (i) *T*_1_-weighted gradient echo sequence (with a weak *T*_2_*-weighting), echo time TE 3 ms, repetition time TR 100 ms, flip angle 80°, matrix 128 × 256, field of view FOV 25 × 50 mm, slice thickness 1 mm, and number of acquisitions NA 32; (ii) a strongly *T*_2_*-weighted gradient echo sequence, TE 8 ms, TR 400 ms, flip angle 60°, NA 8, and the same geometry. A relative signal intensity was calculated as a ratio of the signal in the given organ (liver, spleen, kidney) and the signal in the thigh muscle, which was less vascularized and without substantial nanoparticle deposition. Mice were anesthetized by passive inhalation of isoflurane in air (3% for induction and 1.5–2% for maintenance) for both administration of the nanoparticles and MRI examination.

The animal experiments were performed in accordance with national and international guidelines for laboratory animal care and were approved by the Laboratory Animal Care and Use Committee of the First Faculty of Medicine, Charles University, and the Ministry of Education, Youth and Sports of the Czech Republic (MSMT-34384/2019-2).

### 2.10. In Vitro and In Vivo CT Investigation and Imaging

X-ray attenuation of aqueous dispersions of Gd(Tb)F_3_:Tb^3+^(Gd^3+^),Yb^3+^,Nd^3+^@P(DMA-AGME)-Ale nanoparticles (0–35 mg/mL) doped with different concentrations of Gd^3+^, Tb^3+^, Nd^3+^, and Yb^3+^ ions and in vivo CT imaging of mice (CD1) were done using a precli-nical Albira imager (Bruker BioSpin; Ettlingen, Germany). Mice were monitored prior to contrast injection with the following settings: CT bed 125 mm, 45 kV, 400 μA, high quality mode, time >1 h with two rotations of gantry (each 1000 projections). 3D images were reconstructed by filtered back projection with voxel size of 0.125 mm. GdF_3_:Tb^3+^,Yb^3+^,Nd^3+^@P(DMA-AGME)-Ale nanoparticles (120 μL, 27 mg/mL of PBS) were administered retro-orbitally to mice under isoflurane anesthesia (1.5%, 1.5 mL/min) and then again after 24 h. Mice were measured 48 h after the first administration of the particles and the resulting images were compared. Volume of the liver and kidneys was calculated in terms of average X-ray attenuation values (Hounsfield units HU). 

### 2.11. In Vivo Optical Imaging 

Biodistribution of GdF_3_:Tb^3+^,Yb^3+^,Nd^3+^@P(DMA-AGME)-Ale-Cy7 nanoparticles (120 µL of dispersion; 27 mg of particles per mL of PBS) was determined by in vivo fluorescence imaging in two CD1 mice scanned before, immediately after, and 2, 5, 24, 96, and 168 h after retroorbital injection. The mice were shaved on the left side of the body before scanning and anesthetized by 3% isoflurane (2% isoflurane to maintain anesthesia). Fluorescence intensity was measured by an optical Xtreme in vivo imager (Bruker; Ettlingen, Germany) using the excitation filter 750 nm and the emission filter 830 nm. After the measurement, mice were sacrificed by cervical dislocation, selected organs (liver, kidneys, lungs, spleen, and heart) were removed and measured for fluorescence intensity using the same settings. The greyscale adjustments were performed using open source image processing software ImageJ, v. 1.52p (National Institutes of Health; Bethesda, MD, USA).

## 3. Results and Discussion

### 3.1. Synthesis of Gd(Tb)F_3_:Tb^3+^(Gd^3+^),Yb^3+^,Nd^3+^@P(DMA-AGME)-Ale Nanoparticles

Gd(Tb)F_3_:Tb^3+^(Gd^3+^),Yb^3+^,Nd^3+^ nanoparticles were obtained by a one-step coprecipitation of lanthanide salts using EG solvent and water-soluble P(DMA-AGME)-Ale copolymer as a capping agent to control the morphology and colloidal stability. In the system, Gd^3+^ served as *T*_1_-weighted MRI contrast agent, while Tb^3+^ with a short electronic relaxation time enhanced *T*_2_ relaxation. Co-doping of Tb^3+^ with Yb^3+^ was selected to shift the up-conversion emission under 980 nm, while doping with paramagnetic Nd^3+^ ions was chosen to absorb photons at 808 nm. Advantage of poly(*N*,*N*-dimethylacrylamide) (PDMA) coating consisted in that it has been already successfully used in biomedicine, including MRI and drug delivery systems, due to its excellent hydrophilicity and biocompatibility [29,31,32]. Last but not least, *N*,*N*-dimethylacrylamide (DMA) is easy to copolymerize with various functional comonomers to introduce reactive groups for subsequent attachment of biomolecules. DMA was copolymerized with AGME, methyl ester groups of which can be easily transformed to hydrazone, commonly used pH-sensitive linkage for attachment of anticancer drug (e.g., doxorubicin). P(DMA-AGME)-Ale copolymer was synthesized by a simple radical copolymerization [29]. Preliminary experiments showed that the concentration of P(DMA-AGME)-Ale in EG has to be >10^−2^ mg/mL to obtain nanoparticles stable in water and/or PBS. In comparison, P(DMA-AGME)-coated nanoparticles prepared in water precipitated immediately after transfer in water or PBS. However, size, dispersity, and colloidal stability of GdF_3_ nanoparticles is known to be controlled by a combination of a stabilizing agent (e.g., PSSMA) and EG [8,33]. To investigate the effect of the stabilizer concentration in EG on colloidal stability of the Gd(Tb)F_3_-based nanoparticles, they were prepared with different amounts of PDMA of various molar mass (Table S1; Supporting Data). Increasing concentrations of polymer in EG increased the colloidal stability of particles. Preferred *M_w_* of PDMA reached 8000 or 1.1 × 10^4^ g/mol to efficiently stabilize the nanoparticles in buffer solutions, which agreed with literature data [29]. PDMA of *M_w_* = 1.1 × 10^4^ g/mol was then selected for all other experiments in this study.

**Figure 2:**
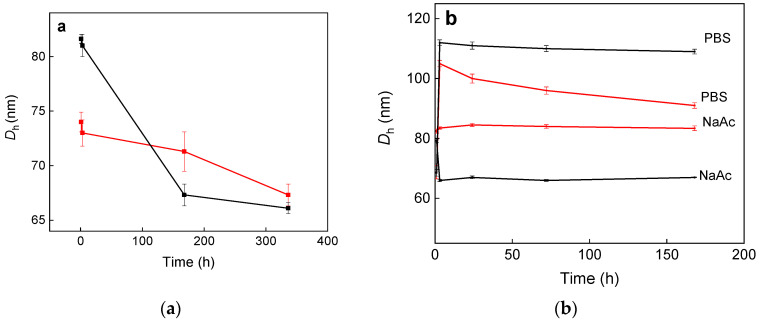
Dependence of hydrodynamic diameter *D*_h_ of P(DMA-AGME)-Ale-coated GdF_3_:Tb^3+^,Yb^3+^,Nd^3+^ (red) and TbF_3_:Gd^3+^,Yb^3+^,Nd^3+^ nanoparticles (black) in (**a**) water and (**b**) PBS and NaAc buffers. The particles were prepared at reaction temperature 75 °C.

Hydrodynamic size of P(DMA-AGME)-Ale-coated GdF_3_:Tb^3+^,Yb^3+^,Nd^3+^ and TbF_3_:Gd^3+^,Yb^3+^,Nd^3+^ nanoparticles in water was 74 and 82 nm, respectively, with a rather narrow polydispersity *PD* ~0.075, (Table 1, Figure 2a). Narrow particle size distribution is important in terms of controllable physicochemical and biological properties and reproducibility of the results [34]. ζ-potential of the nanoparticles was slightly positive (1–2 mV), which could support their internalization in the cells that are typically negatively charged. Hydrodynamic size of both particle types in PBS and sodium acetate (NaAc) buffers was recorded also as a function of time (Figure 2b). The nanoparticle size increased by 30–40 nm after 24 h in PBS and then remained constant during two weeks without any sign of sedimentation. Moreover, coating with P(DMA-AGME)-Ale colloidally stabilized the Gd(Tb)F_3_:Tb^3+^(Gd^3+^),Yb^3+^,Nd^3+^ nanoparticles in NaAc buffer for more than month without any sign of aggregation. Investigation of the effect of the reaction temperature on the colloidal stability of the particles revealed that the hydrodynamic dia-meters of GdF_3_:Tb^3+^,Yb^3+^,Nd^3+^@P(DMA-AGME)-Ale nanoparticles increased with increasing temperature from 75 to 140 °C, documenting worsening of the colloidal stability (Figure S1). Colloidal instability of the particles synthesized at 140 °C was observed in PBS (pH = 7.4; *D*_h_ = 791 nm, *PD* = 0.19), while the particles synthesized at 100 °C were stable for one week (*D*_h_ = 244 nm, *PD* = 0.2).

**Figure 3:**
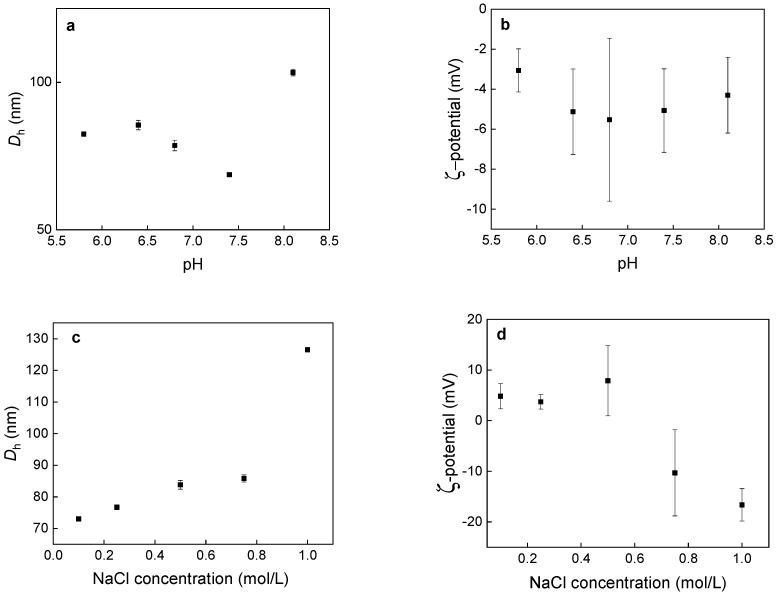
Dependence of (**a**,**c**) hydrodynamic diameter *D*_h_ and (**b**,**d**) ζ-potential of GdF_3_:Tb^3+^,Yb^3+^,Nd^3+^@P(DMA-AGME)-Ale nanoparticles on (**a**,**b**) pH and (**c**,**d**) NaCl concentration.

To further investigate colloidal stability of GdF_3_:Tb^3+^,Yb^3+^,Nd^3+^@P(DMA-AGME)-Ale nanoparticles, their hydrodynamic size and ζ-potential were measured at different ionic strengths of NaCl solutions and different pHs of PBS (Figure 3). No significant size and ζ-potential changes were observed in PBS. Slightly negative ζ-potential of the particles (−3 till −5 mV) in all series of PBS can be explained by suppression of particle charges by ionic strength of PBS. In the dependence of *D*_h_ of the particles on concentration of NaCl, the size slightly increased at concentration of 1 mol/L due to partial particle aggregation (Figure 3c). Let us note that no significant increase of hydrodynamic particle size was observed at biological concentration of NaCl (100–150 mM) [35]. Colloidal stability of the particles was also studied by the evolution of ζ-potential with increasing ionic strength (Figure 3d). However, the absolute value of the ζ-potential increased with increasing NaCl concentration probably due to partial particle aggregation as confirmed in the dependence of the hydrodynamic particle diameter on NaCl concentration (Figure 3c). The surface charge of nanoparticles in biological media is a crucial aspect for many potential applications. It can facilitate in vivo circulation of negatively charged particles in the body or support uptake of positively charged particles by the cells [36]. 

**Figure 4:**
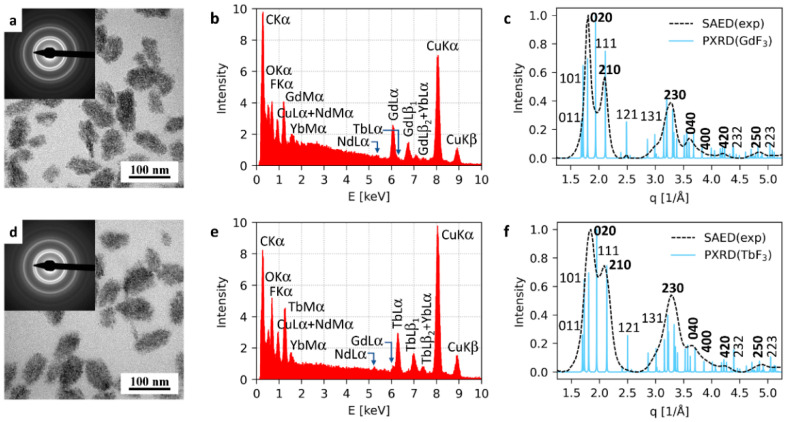
Characterization of P(DMA-AGME)-Ale-coated (**a**–**c**) GdF_3_:Tb^3+^,Yb^3+^,Nd^3+^ and (**d**–**f**) TbF_3_:Gd^3+^,Yb^3+^,Nd^3+^ nanoparticles. (**a**,**d**) TEM micrographs of the nanoparticles with insets showing their diffraction patterns, (**b**,**e**) TEM/EDX spectra, and (**c**,**f**) comparison of experimental selected electron diffraction patterns (TEM/SAED) with calculated powder X-ray diffraction patterns (PXRD) of orthorhombic GdF_3_ and TbF_3_ structures; the *hk*0 diffractions, which were stronger in TEM/SAED than in PXRD due to preferred orientation of nanocrystals, are marked with bold font.

The morphology, size, and size distribution of P(DMA-AGME)-Ale-coated GdF_3_:Tb^3+^,Yb^3+^,Nd^3+^ and TbF_3_:Gd^3+^,Yb^3+^,Nd^3+^ nanoparticles prepared in EG were analyzed by TEM (Figure 4). The small crystalline nanoparticles with sizes <5 nm (high magnification TEM micrographs are shown in Figure S2) tended to form 51 nm and 59 nm aggregates, respectively (Figure 4a,d; Table 1) with a rather low dispersity (*Ð* ~1.15). The elemental analysis (TEM/EDX; Figure 4b,e) confirmed the expected elemental composition, including low concentration of ions in GdF_3_:Tb^3+^,Yb^3+^,Nd^3+^ and TbF_3_:Gd^3+^,Yb^3+^,Nd^3+^ particles; the strong peaks of C and Cu originated from the supporting carbon-coated copper grids, onto which the nanoparticles were deposited. The comparison of experimental selected-area electron diffraction (TEM/SAED) with theoretically calculated powder X-ray diffraction patterns (PXRD) confirmed that the crystalline structures corresponded to orthorhombic GdF_3_ and TbF_3_ modifications (Figure 4c,f). The positions of TEM/SAED diffractions corresponded precisely to the positions of theoretically calculated PXRD diffractions. However, the experimental TEM/SAED diffraction intensities were somewhat different from the calculated PXRD intensities. The reason is that PXRD patterns were calculated for the random orientation of the crystals, while our results suggested that both GdF_3_ and TbF_3_ formed thin flat nanoplatelets that tended to lay on the carbon film on their small facets oriented in such a way that their shortest unit cell parameter was parallel with the electron beam. This corresponded to the preferred orientation of the nanocrystals with zone axis [*uvw*] = [001]. According to Weiss zone law (WZL: *hu* + *kv* + *lw* = 0, where *h*, *k*, *l* are diffraction indexes and *u*, *v*, *w* are the indexes of the zone axis [37], the strongest diffractions should be of the type [*hk*0], i.e., their last diffraction index should be zero (because in our case the WZL takes a simple form: *hu* + *kv* + *lw* = *h*0 + *k*0 + *l*1 = *l* = 0). This was in perfect agreement with the TEM/SAED results, as for both GdF_3_ (Figure 4c) and TbF_3_ (Figure 4f), the *hk*0 SAED diffractions were stronger than corresponding PXRD diffractions, while the intensive *hkl* PXRD diffractions exhibited almost negligible intensity in the experimental SAED patterns.

FTIR spectroscopy and TGA characterized the P(DMA-AGME)-Ale-coated Gd(Tb)F_3_:Tb^3+^(Gd^3+^),Yb^3+^,Nd^3+^ nanoparticle surface (Figure S3). Successful modification of the nanoparticles was documented by the FTIR spectra, where characteristic P(DMA-AGME)-Ale peaks included absorption band at 1620 cm^−1^ ascribed to *ν*(C=O) stretching vibrations of the amide groups and peaks at 2925 and 2860 cm^−1^ were attributed to *ν_as_*(CH_3_) asymmetric and *ν_s_*(CH_2_) symmetric stretching vibrations (Figure S3a). Peak at 1745 cm^−1^ was ascribed to *ν*(C=O) stretching vibrations of the ester group of *N*-acryloylglycine methylester. According to TGA of P(DMA-AGME)-Ale-coated TbF_3_:Gd^3+^,Yb^3+^,Nd^3+^ and GdF_3_:Tb^3+^,Yb^3+^,Nd^3+^ particles, amount of P(DMA-AGME)-Ale polymer on the surface was 8.3 and 5.3 wt.%, respectively (Figure S3b). Weight losses (~6.5 wt.%) observed upon heating from RT to ~300 °C were ascribed to evaporation of water and EG. 

### 3.2. Down- and Upconversion Luminescence of Gd(Tb)F_3_:Tb^3+^(Gd^3+^),Yb^3+^,Nd^3+^@P(DMA-AGME)-Ale Nanoparticles

**Figure 5:**
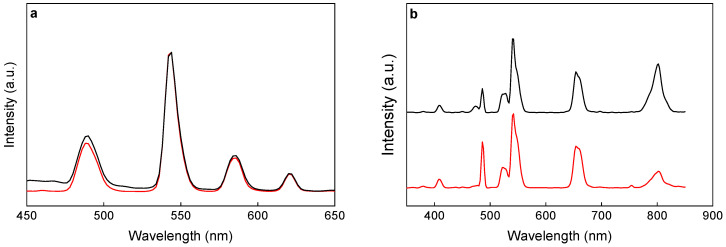
(**a**) DC and (**b**) UC photoluminescence emission spectra of P(DMA-AGME)-Ale-coated GdF_3_:Tb^3+^,Yb^3+^,Nd^3+^ (red) and TbF_3_:Gd^3+^,Yb^3+^,Nd^3+^ nanoparticles (black) excited at (**a**) 272 nm (for GdF_3_:Tb^3+^) or 350 nm (for TbF_3_:Gd^3+^) and (**b**) 980 nm; particle concentration 1 mg/mL and power density of up-conversion luminescence 5 W/cm^2^.

Luminescence properties of P(DMA-AGME)-Ale-coated Gd(Tb)F_3_:Tb^3+^(Gd^3+^),Yb^3+^,Nd^3+^ nanoparticles were determined by the excitation and emission spectra (Figure 5, Figures S3 and S4). The excitation spectra of Tb^3+^- and Gd^3+^-containing nanoparticles exhibited the downconversion emission of Tb^3+^ due to ^5^D_4_ → ^7^F_5_ transition at 544 nm (Figure S4a). The excitation peaks at 284 (^5^I_6_), 302 (^5^H_6_), 318 (^5^D_0_), 340 (^5^G_2_), 350 (^5^D_2_), 368 (^5^G_6_), 378 (^5^D_3_), and 484 (^5^D_4_) nm originated from the transitions of ^7^F_6_ ground state to different excited states of Tb^3+^ [38]. The excitation peak at 272 nm was ascribed to ^8^S_7/2_ → ^6^I_7/2_ transition of Gd^3+^ [8]. Upon excitation at 272 (for GdF_3_:Tb^3+^) and 350 nm (for TbF_3_:Gd^3+^), the emission spectrum of the nanoparticles displayed blue, green, and red bands attributed to ^5^D_4_ → ^7^F_6_ (490 nm), ^5^D_4_ → ^7^F_5_ (544 nm), ^5^D_4_ → ^7^F_4_ (586 nm), and ^5^D_4_ → ^7^F_3_ (620 nm) transitions of the characteristic Tb^3+^ emission (Figure 5a). It is important that the TbF_3_-based nanoparticles can be excited by almost all wavelengths in the range of 250–400 nm (Figure S4b).

Unique spectral properties of Tb^3+^-doped UC nanocrystals can offer several simultaneous emissions compared to one emission of conventional biolabels, since the proportion of blue, green, and red emissions radiating from the same ^5^D_4_ state is unchanged for all pump powers. Any spectral differences induced by living tissues can be used to probe their scattering and absorption coefficients [39]. Both Tb^3+^ and Nd^3+^ up-conversion emission was observed under 980 nm excitation (Figure 5b). The upconversion emission spectra of the nanoparticles displayed green, yellow, and bands attributed to ^4^G_9/2_ → ^4^I_9/2_ (520–530 nm), ^4^G_9/2_ → ^4^I_13/2_/^4^G_7/2_ → ^4^I_11/2_ (555 nm), and ^4^F_5/2_ → ^4^I_9/2_ (790–820 nm) transitions of the characteristic Nd^3+^ emissions [20] and bands corresponding to ^5^D_3_ → ^7^F_5_ (408 nm), ^5^D_4_ → ^7^F_6_ (488 nm) and ^5^D_4_ → ^7^F_5_ (542 nm) transitions of Tb^3+^ [40]. The upconversion spectra of P(DMA-AGME)-Ale-coated GdF_3_:Tb^3+^,Yb^3+^,Nd^3+^ and TbF_3_:Gd^3+^,Yb^3+^,Nd^3+^ nanoparticles synthesized under the same conditions but with different concentrations of Tb^3+^ ions (Figure S5) showed that cooperative emission from a pair of excited Yb^3+^ ions strongly depended on Tb^3+^ content, which agreed with literature data [41]. The emission from Tb^3+^, caused by cooperative energy transfer (CET) from Yb^3+^, was clearly observed in each sample. The peak intensities at 488 nm increased with increasing Tb^3+^ concentration indicating that upconversion luminescence of the Yb^3+^–Yb^3+^ pair was involved in CET. Enhanced Tb^3+^ doping led to a decrease of CET efficiency of Tb^3+^ to Yb^3+^ ions, as the average distance decreased, which lowered intensity of Tb^3+^ emissions. As a result, the emission band at 488 nm originated not directly from the Yb^3+^–Yb^3+^ pair, but from Tb^3+^ at low doping concentration. Furthermore, the emission of Nd^3+^ at 796 nm and dominant green emission of Tb^3+^ at 542 nm were detected for both P(DMA-AGME)-Ale-coated GdF_3_:Tb^3+^,Yb^3+^,Nd^3+^ (10% Tb) and TbF_3_:Gd^3+^,Yb^3+^,Nd^3+^ nanoparticles (70% Tb). In the spectra of the other particles (0, 20, 40, 50 and 90% Tb), the emission intensity of Tb^3+^ and Nd^3+^ was rather low.

### 3.3. Cytotoxicity of Gd(Tb)F_3_:Tb^3+^(Gd^3+^),Yb^3+^,Nd^3+^@P(DMA-AGME)-Ale Nanoparticles

As the main goal in the design of new nanoparticles is to use them as a multimodal imaging agent in various bioapplications, it is crucial to assess their biocompatibility. The PrestoBlue™ viability tests after incubation of the HeLa and HF cells with P(DMA-AGME)-Ale-coated both GdF_3_:Tb^3+^,Yb^3+^,Nd^3+^ and TbF_3_:Gd^3+^,Yb^3+^,Nd^3+^ nanoparticles showed similar trends (Figure S6). The nanoparticles (1 mg/mL) were not cytotoxic for HF cells, but the higher concentration (2 mg/mL) significantly decreased viability to 80%. The viability of HeLa cells slightly, but nonsignificantly, decreased at particle concentrations of 0.5 and 1 mg/mL and it decreased significantly (to 40%) at 2 mg/mL. The TbF_3_:Gd^3+^,Yb^3+^,Nd^3+^ nanoparticles were slightly more cytotoxic for HeLa cells than the GdF_3_:Tb^3+^,Yb^3+^,Nd^3+^ ones at the highest concentration. However, the viability HeLa cells in the presence of nanoparticles at the highest concentration (2 mg/mL) was lower (~40%) than that of HF cells (80%). The difference in viability between the cells can be induced by at least twice faster proliferation rate of HeLa cells compared to HF cells, which are primary slowly proliferating cells. The faster growing HeLa cells are thus more sensitive to the cytotoxic effect of the nanoparticles. Strategies to reduce unwanted side-effects of the particles at high concentration are in progress, including their encapsulation by another polymer, grafting of lanthanide chelates on the particles, ligand exchange, etc.

### 3.4. Intracellular Uptake of Gd(Tb)F_3_:Tb^3+^(Gd^3+^),Yb^3+^,Nd^3+^@P(DMA-AGME)-Ale Nanoparticles Determined by Laser Scanning Confocal Microscopy

**Figure 6:**
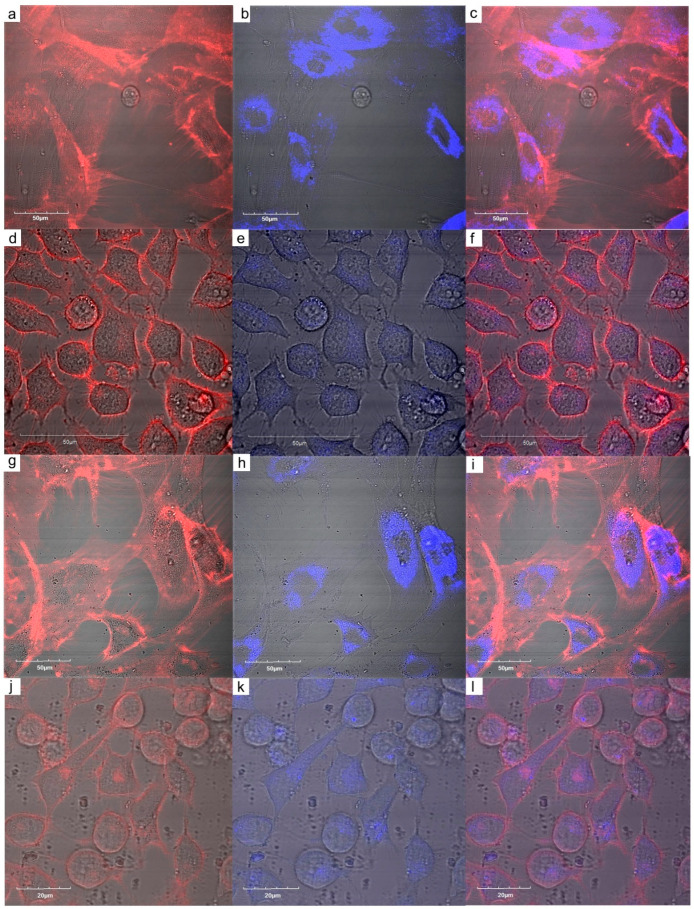
Intracellular uptake of (**a**–**f**) TbF_3_:Gd^3+^,Yb^3+^,Nd^3+^@P(DMA-AGME)-Ale and (**g**–**l**) GdF_3_:Tb^3+^,Yb^3+^,Nd^3+^@P(DMA-AGME)-Ale nanoparticles by (**d**–**f**,**j**–**l**) Hela and (**a**–**c**,**g**–**i**) HF cells. (**a**,**d**,**g**,**j**) CellMask™ deep red-stained cell membranes were visualized in red channel and (**b**,**e**,**h**,**k**) nanoparticles were visualized in blue channel. (**c**,**f**,**i**,**l**) Both channels were merged.

Hela and HF cells were incubated for 24 h with Gd(Tb)F_3_:Tb^3+^(Gd^3+^),Yb^3+^,Nd^3+^@P(DMA-AGME)-Ale nanoparticles that were visualized intracellularly (Figure 6). There was a strong difference in intensity of particle signal between HeLa (Figure 6d–f,j–l) and HF cells (Figure 6a–c,g–i) in confocal micrographs, which could be caused by the different uptake of primary and cancer cells. The other fact that can also play a role consists in different accumulation of the nanoparticles in HF and HeLa cells. As already stated above, the proliferation rate of HF cells is significantly slower than that of HeLa cells and because we used the same particle concentration for treatment of both cell types, in the case of slowly growing HF cells, their number was lesser compared to smaller HeLa cells. For the sake of comparison, laser scanning confocal micrographs of HF and HeLa cells before and after treatment with (Gd)TbF_3_:Tb^3+^(Gd^3+^),Yb^3+^,Nd^3+^@P(DMA-AGME)-Ale nanoparticles were shown in Figure S7. 

**Figure 7:**
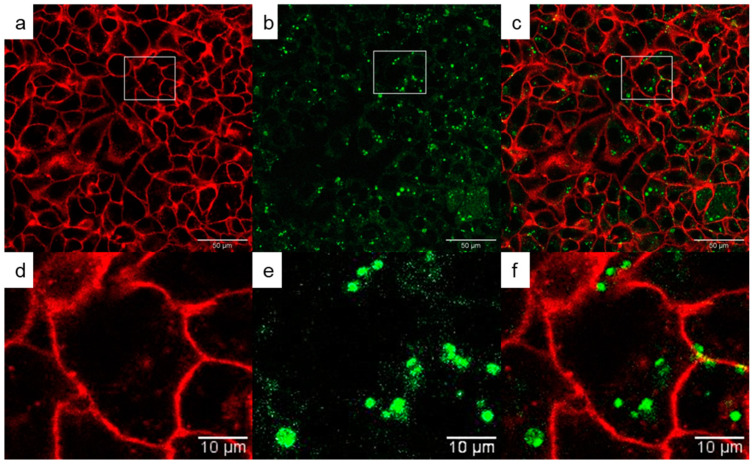
(**a**–**f**) Confocal micrographs of distribution of GdF_3_:Tb^3+^,Yb^3+^,Nd^3+^@P(DMA-AGME)-Ale nanoparticles in HepG2 cells at 808 nm excitation with a laser power of 30–50 mW. (**d**–**f**) Detailed micrographs of (**a**–**c**). (**a**,**d**) CellMask™ deep red-stained cell membrane, (**b**,**e**) nanoparticles (green), and (**c**,**f**) overlay of (**a**,**b**) and (**d**,**e**), respectively.

In investigation of biodistribution of the particles within the cells, their further two types were used, HepG2 (cancer cells of human liver tumor) and INS-1E cells from rat insulinoma, which is a tumor of the pancreas derived from beta cells secreting insulin. In vitro labeling of living HepG2 and INS cells after 24 h of incubation with upconverting Gd(Tb)F_3_:Tb^3+^(Gd^3+^),Yb^3+^,Nd^3+^@P(DMA-AGME)-Ale nanoparticles was demonstrated by confocal microscopy at 808 nm pulsed excitation with 140 fs (Figure 7 and Figure S8); cell membranes were stained with CellMask™ deep red. The particles were engulfed by the cells already after 2 h of incubation; however, 24-h incubation was selected in further experiments as it ensured complete internalization. Comparing of the emission spectra of neat nanoparticles with those in cells (Figure S9) showed the nanoparticles localized in the HepG2 and INS cells as demonstrated within the *xy* confocal plane. The particles were distributed in the cell cytosol. 

### 3.5. MR Relaxometry of Gd(Tb)F_3_:Tb^3+^(Gd^3+^),Yb^3+^,Nd^3+^@P(DMA-AGME)-Ale Nanoparticles

Nanoparticle relaxivities of aqueous Gd(Tb)F_3_:Tb^3+^(Gd^3+^),Yb^3+^,Nd^3+^@P(DMA-AGME)-Ale nanoparticle dispersions were summarized in the Table 2. While the whole crystal structure contributed to high *T*_2_ relaxation, *T*_1_ relaxation depended predominantly on Gd^3+^ content in the nanoparticles. If Gd^3+^ was absent, the *r*_1_ relaxivity was very low, as other lanthanide ions (Nd^3+^, Tb^3+^, Yb^3+^) had negligible effect on *T*_1_ relaxation time compared to Gd^3+^ (data not shown). Nevertheless, even a small amount of Gd^3+^ in the crystal significantly increased *r*_1_ relaxivity. Interestingly, particles without Gd^3+^ had a high *r*_2_*/r*_1_ ratio due to very low *r*_1_, while their *r*_2_ remained comparable to that of Gd-containing particles. The MR relaxivity was comparable to that of commercial contrast agents based on gadolinium chelates, such as Gd-DTPA (*r*_1_ = 3.7 mM^−1^ s^−1^ and *r*_2_ = 5.8 mM^−1^ s^−1^). Somewhat smaller relaxivity of the Gd(Tb)F_3_:Tb^3+^(Gd^3+^),Yb^3+^,Nd^3+^@P(DMA-AGME)-Ale nanoparticles (per Ln^3+^ ions) can be explained by lower accessibility of inner ions, which lowered water exchange as a main contributor to *r*_1_. Interestingly, the particles with low Gd content revealed higher *r*_1_ relaxivity than expected with respect to Gd concentration. Doping of Yb^3+^, Nd^3+^, and Tb^3+^ ions into GdF_3_ nanoparticles contributed to relaxivity mechanisms and provided facile strategy for synthesis of new *T*_1_/*T*_2_ dual-weighted contrast Ln^3+^-based agents, which agreed with literature [22].

### 3.6. In Vivo MR Imaging of Gd(Tb)F_3_:Tb^3+^(Gd^3+^),Yb^3+^,Nd^3+^@P(DMA-AGME)-Ale Nanoparticles 

**Figure 8:**
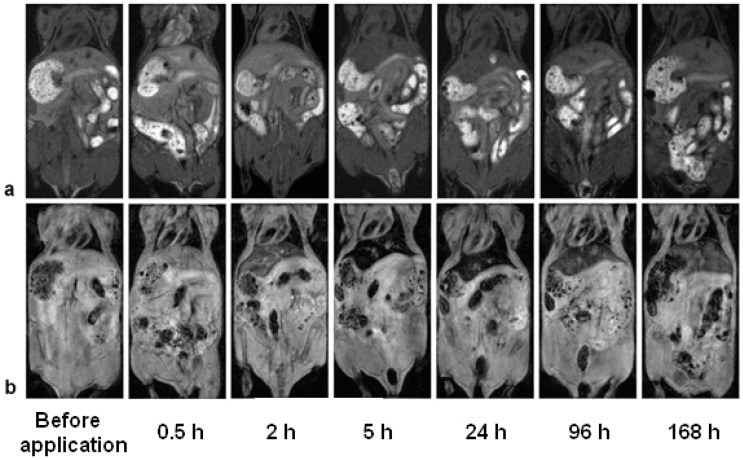
(**a**) *T*_1_- and (**b**) *T*_2_*-weighted MR images of the mouse before retroorbital administration of TbF_3_:Gd^3+^,Yb^3+^,Nd^3+^@P(DMA-AGME)-Ale nanoparticles and in several time intervals after the administration. (**a**) The signal in the liver on the *T*_1_-weighted image moderately increased shortly after the administration due to a weak *T*_1_ effect, while (**b**) the signal on the *T*_2_*-weighted MR image of the liver and spleen substantially decreased due to nanoparticle accumulation in the organs. The nanoparticles were slowly excreted from the liver after one day of post-administration; however, signal hypointensity was noticeable even one week after the administration.

Gd(Tb)F_3_:Tb^3+^(Gd^3+^),Yb^3+^,Nd^3+^@P(DMA-AGME)-Ale nanoparticles were investigated in vivo as a *T*_1_- and *T*_2_-weighted MRI contrast agent. When TbF_3_:Gd^3+^,Yb^3+^,Nd^3+^@P(DMA-AGME)-Ale nanoparticles (with low *r*_1_ and high *r*_2_ relaxivity) were administered into the blood stream of experimental mice, a small increase of the signal was immediately visible on *T*_1_-weighted images in the liver (Figure 8a). Signal darkening after 5 h might be explained by a weak *T*_2_* effect, which probably outweighed the *T*_1_ effect, as the nanoparticles slowly accumulated (and possibly aggregated) in the liver. This hypothesis was supported by *T*_2_*-weighted images (Figure 8b). Accumulation of the nanoparticles was manifested by a strong hypointense signal in the liver and spleen with a maximum between 5 and 24 h after the administration. Then, slow recovery of the signal was observed, which corresponded to a gradual excretion of the nanoparticles from the organism.

**Figure 9:**
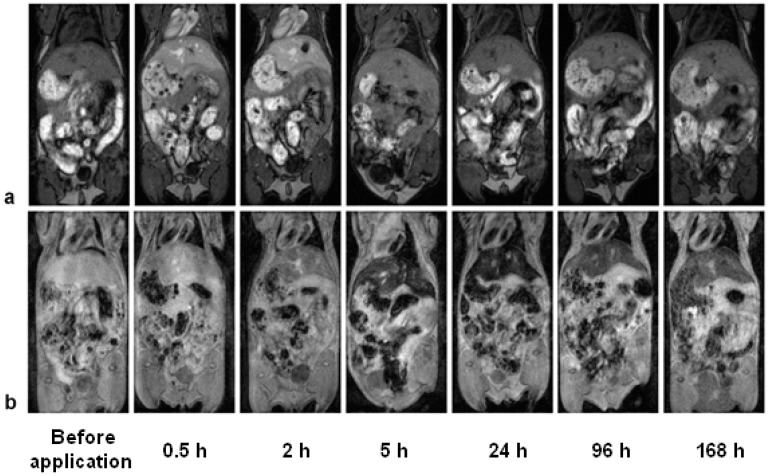
(**a**) *T*_1_-weighted and (**b**) *T*_2_*-weighted MR images of the mouse before retroorbital admi-nistration of GdF_3_:Tb^3+^,Yb^3+^,Nd^3+^@P(DMA-AGME)-Ale nanoparticles and in several time intervals after the administration. (**a**) The signal in the liver on the *T*_1_-weighted image substantially increased shortly after the administration due to a strong *T*_1_ effect, while (**b**) the signal on the *T*_2_*-weighted MR image in the liver and spleen decreased due to nanoparticle accumulation in the organs. Slow excretion of the nanoparticles from the liver was observed after one day post-admi-nistration.

Gd^3+^ ions in the nanoparticles enhanced the *T*_1_-weighted MRI of mice. Application of GdF_3_:Tb^3+^,Yb^3+^,Nd^3+^@P(DMA-AGME)-Ale nanoparticles with high *r*_1_ and *r*_2_ relaxivities caused a hyperintense signal on *T*_1_-weighted images in the liver, spleen (Figure 9a), and kidneys immediately after the administration. *T*_2_*-weighted images (Figure 9b) revealed a strong hypointense signal in the liver and spleen, again delayed by several hours. The *T*_2_* signal in the kidneys was not significantly affected. 

Relative signal evolution after administration of Gd(Tb)F_3_:Tb^3+^(Gd^3+^),Yb^3+^,Nd^3+^@P(DMA-AGME)-Ale nanoparticles in the mice was shown in Figure S10. Small changes were observed only in *T*_1_ signal after TbF_3_:Gd^3+^,Yb^3+^,Nd^3+^@P(DMA-AGME)-Ale administration (Figure S10a), while application of GdF_3_:Tb^3+^,Yb^3+^,Nd^3+^@P(DMA-AGME)-Ale particles caused a substantial *T*_1_ signal increase in the liver, spleen, and kidneys shortly after the administration, which was followed by a drop back to original values within 5 h (Figure S10c). Relative *T*_2_* signal dropped in the liver and spleen within 5 h after application of both TbF_3_:Gd^3+^,Yb^3+^,Nd^3+^ (Figure S10b) and GdF_3_:Tb^3+^,Yb^3+^,Nd^3+^ nanoparticles (Figure S10d). Recovery to normal values started 24 h post injection and was very slow, the signal was lowered even after one week. No substantial change of *T*_2_* was observed in the kidneys.

The observed time delay between the maximum of the hyperintense signal on *T*_1_-weighted images and minimum of the hypointense signal on *T*_2_*-weighted images might reflect redistribution of the nanoparticles in the organism (Figure 8 and Figure 9 and Figure S10). We hypothesize that the initial hyperintense signal on *T*_1_-weighted images corresponded rather to nanoparticle circulation in the blood vessels, while late onset of hypointensities on *T*_2_*-weighted images reflected accumulation of the nanoparticles in the liver and spleen tissue, and possibly also particle aggregation, leading to a stronger *T*_2_* effect.

### 3.7. In Vitro and In Vivo CT Investigation and Imaging of Gd(Tb)F_3_:Tb^3+^(Gd^3+^),Yb^3+^,Nd^3+^@P(DMA-AGME)-Ale Nanoparticles 

Capability of Gd(Tb)F_3_:Tb^3+^(Gd^3+^),Yb^3+^,Nd^3+^@P(DMA-AGME)-Ale nanoparticles as a CT contrast agent was evaluated at different concentrations and constant energy of 45 keV (Figure S11). Enhanced signals of the CT images were observed with the increasing concentration of the particles. While the CT value of water amounted to 3 Hounsfield units (HU), that of the particles increased linearly with their concentrations (Figure S11). Undoped GdF_3_ nanoparticles and Nd^3+^- or Yb^3+^-doped GdF_3_ showed a weaker signal, while combination of Tb^3+^, Gd^3+^, Nd^3+^, and Yb^3+^ ions in one particle enhanced the signals. Compared to clinically used Iohexol CT contrast agent dispersed in water at different concentrations, Gd(Tb)F_3_:Tb^3+^(Gd^3+^),Yb^3+^,Nd^3+^@P(DMA-AGME)-Ale nanoparticles showed higher X-ray attenuation at the same concentrations due to a higher K-edge energy. In the dependence of CT values on the particle concentration, slopes of the P(DMA-AGME)-Ale-coated TbF_3_:Gd^3+^,Yb^3+^,Nd^3+^ and GdF_3_:Tb^3+^,Yb^3+^,Nd^3+^ nanoparticles were 9.6 and 8.5, respectively, which was more than for clinical Iohexol (4.8). Compared to another commercial agent Iodine, X-ray attenuation of Tb- or Gd-based fluorides was quite similar to those published previously [42,43]. Our lanthanide nanoparticles thus show a great promise as a novel CT contrast agent that is expected to have a superior performance in clinical practice. 

**Figure 10:**
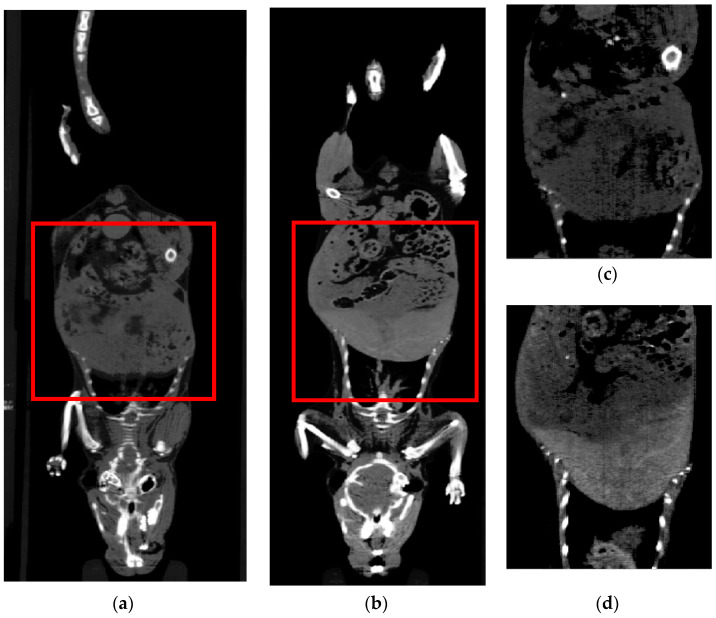
Coronal CT images of the mouse (**a**,**c**) before and (**b**,**d**) after administration of GdF_3_:Tb^3+^,Yb^3+^,Nd^3+^@P(DMA-AGME)-Ale nanoparticles; (**c**,**d**) enlarged CT view of the abdomen.

Nanoparticles with high atomic number of elements can absorb the X-rays to distinguish organs and bones in the CT diagnostics. For in vivo CT imaging, mice were scanned before and after the injection of GdF_3_:Tb^3+^,Yb^3+^,Nd^3+^@P(DMA-AGME)-Ale nanoparticles with the best X-ray attenuation value. To investigate in vivo applicability of the GdF_3_:Tb^3+^,Yb^3+^,Nd^3+^@P(DMA-AGME)-Ale nanoparticles (3 mg/day) as a long-term contrast agent in CT imaging, they were twice retro-orbitally administered in mice. CT images of pre-injected mice (Figure 10a,c) and mice after two particle injections (Figure 10b,d) were compared in coronal projection slices, displaying an enhanced CT signal in the liver and kidneys after second injection of the particles (i.e., 48 h after beginning of the experiment). CT values for the liver and kidneys increased to 152 and 143 HU from original 102 and 119 HU, respectively (Figure S12), suggesting that the GdF_3_:Tb^3+^,Yb^3+^,Nd^3+^@P(DMA-AGME)-Ale nanoparticles are useful as a long-term CT contrast imaging agent. 

### 3.8. In Vivo Optical Imaging of GdF_3_:Tb^3+^,Yb^3+^,Nd^3+^@P(DMA-AGME)-Ale-Cy7 Nanoparticles 

**Figure 11:**
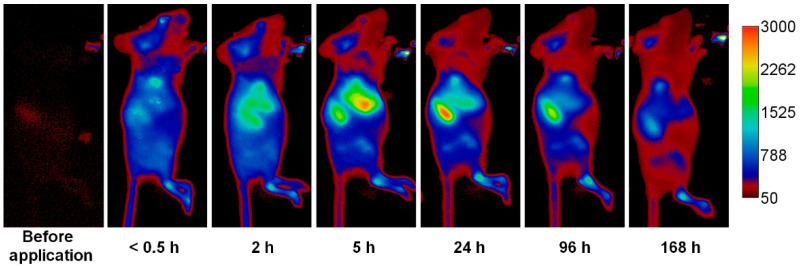
In vivo fluorescence imaging of the mouse after retroorbital administration of GdF_3_:Tb^3+^,Yb^3+^,Nd^3+^@P(DMA-AGME)-Ale-Cy7 nanoparticles.

The GdF_3_:Tb^3+^,Yb^3+^,Nd^3+^@P(DMA-AGME)-Ale nanoparticles were conjugated with the fluorescent dye, sulfocyanine7-Ale (Cy7-Ale), to visualize their in vivo accumulation in the mice body using an Xtreme imaging system. After the conjugation, ξ-potential of the particles decreased from 2 to −5 mV and the hydrodynamic diameter increased from 81 nm (*PD =* 0.02) to 99 nm (*PD =* 0.19). The typical fluorescence of Cy7-modified Gd(Tb)F_3_:Tb^3+^(Gd^3+^),Yb^3+^,Nd^3+^@P(DMA-AGME)-Ale nanoparticles was observed under excitation and emission at 750 and 780 nm, respectively (Figure S13). In vivo optical imaging of mice after administration of the GdF_3_:Tb^3+^,Yb^3+^,Nd^3+^@P(DMA-AGME)-Ale-Cy7 nanoparticles revealed their circulation in the blood, and in particular, slow accumulation in the liver and spleen (Figure 11). The signal in the liver and spleen increased mainly within first 24 h. The nanoparticles were then slowly excreted as manifested by decreasing fluorescence, especially 96 h after administration of the particles. Finally, the mice were sacrificed immediately after the last in vivo scanning; fluorescence imaging revealed presence of the nanoparticles in selected excised mouse organs, mainly in the liver and spleen (Figure S14). It should be noted that Cy7-Ale-conjugated GdF_3_:Tb^3+^,Yb^3+^,Nd^3+^@P(DMA-AGME)-Ale nanoparticles appeared to be chemically stable *in vivo*, as the dye did not desorb from them and was not found in urine, when mouse was monitored 2 h after retroorbital administration. 

## 4. Conclusions

To the best of our knowledge, there are so far no reports on co-doping of fluoride contrast agents with Gd^3+^, Tb^3+^, Yb^3+^, and Nd^3+^ ions to provide DC/UC luminescence, MRI, and CT contrast in one system. Hence, we synthesized GdF_3_ and TbF_3_ nanoparticles doped with Ln^3+^ ions, such as Gd^3+^, Tb^3+^,Yb^3+^, and Nd^3+^, by employing a one-step coprecipitation of respective lanthanide chlorides in EG. Thanks to introduction of hydrophilic P(DMA-AGME)-Ale coating, aqueous dispersions of the nanoparticles were generally highly colloidally stable during weeks till months. In particular, the TbF_3_:Gd^3+^,Yb^3+^,Nd^3+^@P(DMA-AGME)-Ale nanoparticles exhibited down- and upconverting luminescence properties simultaneously with magnetic properties suitable for *T*_2_*-weighted MRI. In contrast, GdF_3_:Tb^3+^,Yb^3+^,Nd^3+^@P(DMA-AGME)-Ale nanoparticles provided sufficient signal in down- and upconversion cellular imaging and contrast enhancement in both *T*_1_- and *T*_2_-weighted MRI and CT. Combination of down- and upconversion luminescence with magnetic properties and X-ray attenuation into a single nanoparticle thus demonstrated feasibility of P(DMA-AGME)-Ale-coated Gd(Tb)F_3_:Tb^3+^(Gd^3+^),Yb^3+^,Nd^3+^ nanoparticles for multimodal imaging. In vitro cell investigations then showed that the particles were efficiently internalized by the primary and cancer cells (HeLa, HF, HepG2, and INS) without entering the nuclei. Distribution of the particles in the cytoplasm was cell type-dependent, they were negligibly cytotoxic for the primary non-cancerogenic cells and more toxic for cancer cells; this is especially important for future in vivo bioimaging of severe medical disorders. For in vivo experiments, a fluorescent dye Cy7 was bound to the Gd(Tb)F_3_:Tb^3+^(Gd^3+^),Yb^3+^,Nd^3+^@P(DMA-AGME)-Ale nanoparticles to enhance fluorescence imaging. The conjugated particles were then trackable by MRI, CT, and FI. The in vivo imaging revealed nanoparticle biodistribution mainly in the liver and spleen of mice. Last but not least, excitation at 808 nm provides opportunity to achieve deeper living tissue penetration and thanks to the upconversion emission at 650 nm, future immobilization of a proper photosensitizer will enable application of P(DMA-AGME)-Ale-coated Gd(Tb)F_3_:Tb^3+^(Gd^3+^),Yb^3+^,Nd^3+^ nanoparticles in photodynamic therapy of tumors. 

## Figures and Tables

**Table 1:** Characterization of the nanoparticles.

Particles	*D*_n_ (nm)	*Ð*	*D*_h_ (nm)	*PD*	ξ-Potential (mV)
GdF_3_:Tb^3+^,Yb^3+^,Nd^3+^ @P(DMA-AGME)-Ale	59	1.16	74	0.08	2
TbF_3_:Gd^3+^,Yb^3+^,Nd^3+^ @P(DMA-AGME)-Ale	51	1.15	82	0.07	1

*D*_n_—number-average particle diameter (TEM); *Ð*—dispersity (TEM); *D*_h_—hydrodynamic diameter (DLS); *PD*—polydispersity (DLS).

**Table 2:** Relaxometry of aqueous Gd(Tb)F_3_:Tb^3+^(Gd^3+^),Yb^3+^,Nd^3+^@P(DMA-AGME)-Ale nanoparticle dispersions measured at 23 °C.

Particles	[Gd^3+^] mmol/mL	[Tb^3+^] mmol/mL	[Yb^3+^] mmol/mL	[Nd^3+^] mmol/mL	∑ [Ln^3+^] mmol/mL	*r*_1_ (mM^−1^ s^−1^)	*r*_2_ (mM^−1^ s^−1^)	*r*_2_/*r*_1_
GdF_3_	143.5	-	-	-	143.5	1.05 ± 0.05	1.27 ± 0.02	1.21
GdF_3_:10%Yb^3+^	133.5	-	16.6	-	150.1	1.41 ± 0.10	1.66 ± 0.02	1.18
GdF_3_:10%Nd^3+^	130.6	-	-	14.0	144.6	1.34 ± 0.05	1.63 ± 0.04	1.22
GdF_3_:5%Yb^3+^,5%Nd^3+^	125.2	-	6.5	9.9	141.6	1.54 ± 0.08	1.86 ± 0.04	1.21
GdF_3_:20%Tb^3+^,5%Yb^3+^,5%Nd^3+^	92.4	27.8	6.9	10.6	137.7	1.29 ± 0.02	1.59 ± 0.03	1.23
GdF_3_:40%Tb^3+^,5%Yb^3+^,5%Nd^3+^	63.2	52.6	6.2	9.5	131.5	1.07 ± 0.01	1.77 ± 0.01	1.65
TbF_3_:40%Gd^3+^,5%Yb^3+^,5%Nd^3+^	58.5	60.2	6.5	10.1	135.3	1.09 ± 0.01	2.34 ± 0.08	2.16
TbF_3_:20%Gd^3+^,5%Yb^3+^,5%Nd^3+^	30.8	103.8	8.2	7.6	150.4	0.69 ± 0.03	1.03 ± 0.02	1.51
TbF_3_:5%Yb^3+^,5%Nd^3+^	-	125.2	7	10.8	143.0	0.04 ± 0.01	2.01 ± 0.01	57.9

## Data Availability

The data presented in this study are available on request from the corresponding author (O.S.).

## Supplementary Materials

The following are available online at https://www.mdpi.com/2079-4991/11/1/230/s1, Figure S1: Dependence of hydrodynamic diameter *D*_h_ of GdF_3_:Tb^3+^,Yb^3+^,Nd^3+^@P(DMA-AGME)-Ale nanoparticles in water on time of storage. The particles were synthesized at 100 or 140 °C. Figure S2: High magnification TEM micrographs of P(DMA-AGME)-Ale-coated (a) GdF_3_:Tb^3+^,Yb^3+^,Nd^3+^ and (b) TbF_3_:Gd^3+^,Yb^3+^,Nd^3+^ nanoparticles. The insets display the very small individual particles with sizes <~5 nm. Figure S3: (a) FTIR spectra and (b) TGA and DTGA (differential thermogravimetric analysis) of P(DMA-AGME)-Ale-coated GdF_3_:Tb^3+^,Yb^3+^,Nd^3+^ (black) and TbF_3_:Gd^3+^,Yb^3+^,Nd^3+^ nanoparticles (red). Figure S4: (a) DC excitation spectra of P(DMA-AGME)-Ale-coated GdF_3_:Tb^3+^,Yb^3+^,Nd^3+^ (red) and TbF_3_:Gd^3+^,Yb^3+^,Nd^3+^ nanoparticles (black); (b) DC emission spectra TbF_3_:Gd^3+^,Yb^3+^,Nd^3+^@P(DMA-AGME)-Ale nanoparticles excited at different wavelengths. Figure S5: UC photoluminescence emission spectra of P(DMA-AGME)-Ale-coated GdF_3_:Tb^3+^,Yb^3+^,Nd^3+^ and TbF_3_:Gd^3+^,Yb^3+^,Nd^3+^ nanoparticles doped with different concentrations of Gd^3+^ and Tb^3+^ ions; 980 nm excitation, particle concentration 1 mg/mL, and power density 5 W/cm^2^. Figure S6: Cytotoxicity of P(DMA-AGME)-Ale-coated GdF_3_:Tb^3+^,Yb^3+^,Nd^3+^ (black) and TbF_3_:Gd^3+^,Yb^3+^,Nd^3+^ nanoparticles (red) incubated with Hela (dashed line) and HF cells (solid line) for 72 h. Figure S7: Laser scanning confocal micrographs of (a–c) HF and (d–f) HeLa cells (a,d) before (negative control) and after treatment with (b,e) TbF_3_:Gd^3+^,Yb^3+^,Nd^3+^@P(DMA-AGME)-Ale and (c,f) GdF_3_:Tb^3+^,Yb^3+^,Nd^3+^@P(DMA-AGME)-Ale nanoparticles. Figure S8: (a–f) Confocal micrographs of biodistribution of TbF_3_:Gd^3+^,Yb^3+^,Nd^3+^@P(DMA-AGME)-Ale nanoparticles in INS cells at 808 nm excitation with a laser power of 30–50 mW. (d–f) Detailed micrographs of (a–c). (a,d) CellMask™ deep red-stained cell membrane, (b,e) nanoparticles (green), and (c,f) overlay of (a,b) and (d,e), respectively. Figure S9: Emission spectra of pure GdF_3_:Tb^3+^,Yb^3+^,Nd^3+^@P(DMA-AGME)-Ale nanoparticles (black) and nanoparticles localized in the HepG2 cells (red). Figure S10: (a,c) Relative *T*_1_ and (b,d) *T*_2_* signal evolution in the liver, kidney (medulla and cortex), and spleen after administration of P(DMA-AGME)-Ale-coated (a,b) TbF_3_:Gd^3+^,Yb^3+^,Nd^3+^ and (c,d) GdF_3_:Tb^3+^,Yb^3+^,Nd^3+^ nanoparticles in mice. Figure S11: Dependence of CT value of Gd(Tb)F_3_:Tb^3+^(Gd^3+^),Yb^3+^,Nd^3+^@P(DMA-AGME)-Ale nanoparticles and Iohexol on the concentration. HU - Hounsfield units. Figure S12: Relative signal evolution of the CT contrast (HU) in the liver and kidneys calculated from pre-injection to 48 h post-injection of GdF_3_:Tb^3+^,Yb^3+^,Nd^3+^@P(DMA-AGME)-Ale nanoparticles. Error bars represent the standard deviation from two independent images. Figure S13: Photoluminescence spectra of Cy7-modified Gd(Tb)F_3_:Tb^3+^(Gd^3+^),Yb^3+^,Nd^3+^@P(DMA-AGME)-Ale nanoparticles. Figure S14: Fluorescence imaging of the excised mouse organs 168 h after administration of GdF_3_:Tb^3+^,Yb^3+^,Nd^3+^@P(DMA-AGME)-Ale-Cy7 nanoparticles. Table S1: Characterization of GdF_3_@PDMAnanoparticles.

## References

[1] Walter A., Paul-Gilloteaux P., Plochberger B., Sefc L., Verkade P., Mannheim J.G., Slezak P., Unterhuber A., Marchetti-Deschmann M., Ogris M. (2020). Correlated multimodal imaging in life sciences: Expanding the biomedical horizon. Front. Phys..

[2] Li X., Zhang X.N., Li X.D., Chang J. (2016). Multimodality imaging in nanomedicine and nanotheranostics. Cancer Biol. Med..

[3] Burke B.P., Cawthorne C., Archibald S.J. (2017). Multimodal nanoparticle imaging agents: Design and applications. Phil. Trans. R. Soc. A.

[4] Key J., Leary J.F. (2014). Nanoparticles for multimodal in vivo imaging in nanomedicine. Int. J. Nanomed..

[5] Rodriguez-Liviano S., Nunez N.O., Rivera-Fernández S., de la Fuente J.M., Ocana M. (2013). Ionic liquid mediated synthesis and surface modification of multifunctional mesoporous Eu:GdF_3_ nanoparticles for biomedical applications. Langmuir.

[6] Wang D.-Y., Ma P.-C., Zhang J.-C., Wang Y.-H. (2018). Efficient down- and up-conversion luminescence in Er^3+^–Yb^3+^ co-doped Y_7_O_6_F_9_ for photovoltaics. ACS Appl. Energy Mater..

[7] Qin X., Zhang X., Zhang W., Li C., Zhu C. (2020). Facile synthesis of NaYF_4_:Ln/NaYF_4_:Eu composite with up-conversion and down-shifting luminescence. J. Photochem. Photobiol. A.

[8] Shapoval O., Kaman O., Hromádková J., Vavřík D., Jirák D., Machová D., Parnica J., Horák D. (2019). Multimodal PSSMA-functionalized GdF_3_:Eu^3+^(Tb^3+^) nanoparticles for luminescence imaging, MRI, and X-ray computed tomography. ChemPlusChem.

[9] Branca M., Pelletier F., Cottin B., Ciuculescu D., Lin C.C., Serra R., Mattei J.G., Casanove M.J., Tan R., Respaud M. (2014). Design of FeBi nanoparticles for imaging applications. Faraday Discuss..

[10] Liang S.Y., Zhou Q., Wang M., Zhu Y.H., Wu Q.Z., Yang X.L. (2015). Water-soluble L-cysteine-coated FePt nanoparticles as dual MRI/CT imaging contrast agent for glioma. Int. J. Nanomed..

[11] Carril M., Fernández I., Rodríguez J., García I., Penadés S. (2013). Gold-coated iron oxide glyconanoparticles for MRI, CT, and US multimodal imaging. Part. Part. Syst. Char..

[12] Alric C., Taleb J., Le Duc G., Mandon C., Billotey C., Le Meur-Herland A., Brochard T., Vocanson F., Janier M., Perriat P. (2008). Gadolinium chelate coated gold nanoparticles as contrast agents for both X-ray computed tomography and magnetic resonance imaging. J. Am. Chem. Soc..

[13] Dong H., Du S.R., Zheng X.-Y., Lyu G.M., Sun L.D., Li L.D., Zhang P.Z., Zhang C., Yan C.H. (2015). Lanthanide nanoparticles: From design toward bioimaging and therapy. Chem. Rev..

[14] Passuello T., Pedroni M., Piccinelli F., Polizzi S., Marzola P., Tambalo S., Conti G., Benati D., Vetrone F., Bettinelli M. (2012). PEG-capped, lanthanide doped GdF_3_ nanoparticles: Luminescent and *T*_2_ contrast agents for optical and MRI multimodal imaging. Nanoscale.

[15] Biju S., Gallo J., Banobre-Lopez M., Manshian B., Soenen S., Himmelreich U., Vander Elst L., Parac-Vogt T. (2018). A magnetic chameleon: Biocompatible lanthanide nanoparticles with magnetic field dependent properties as contrast agents for MRI and optical imaging in biological window. Chem. Eur. J..

[16] Ni D.L., Zhang J.W., Bu W.B., Zhang C., Yao Z., Xing H., Wang J., Duan F., Liu Y., Fan W. (2016). PEGylated NaHoF_4_ nanoparticles as contrast agents for both X-ray computed tomography and ultra-high field magnetic resonance imaging. Biomaterials.

[17] Donati T., Wilson J., Kölbel T., Clough R.E. (2015). Modern diagnostics for type B aortic dissection. Gefasschirurgie.

[18] Viswanathan S., Kovacs Z., Green K.N., Ratnakar S.J., Sherry A.D. (2010). Alternatives to gadolinium-based metal chelates for magnetic resonance imaging. Chem. Rev..

[19] Zhang L., Yang R., Zou H., Shen X., Zheng J., Wei W. (2016). High-efficiency simultaneous three-photon absorption upconversion luminescence of a terbium-doped germanate glass. Jpn. J. Appl. Phys..

[20] Prorok K., Pawlyta M., Stręk W., Bednarkiewicz A. (2016). Energy migration up-conversion of Tb^3+^ in Yb^3+^ and Nd^3+^ codoped active-core/active-shell colloidal nanoparticles. Chem. Mater..

[21] Subramanian M., Thakur P., Gautam S., Chae K.H., Tanemura M., Hihara T., Vijayalakshmi S., Soga T., Kim S.S., Asokan K. (2009). Investigations on the structural, optical and electronic properties of Nd doped ZnO thin films. J. Phys. D.

[22] Yi Z., Li X., Lu W., Liu H., Zeng S., Hao J. (2016). Hybrid lanthanide nanoparticles as a new class of binary contrast agents for in vivo *T*_1_/*T*_2_ dual-weighted MRI and synergistic tumor diagnosis. J. Mater. Chem. B.

[23] Abdesselem M., Schoeffel M., Maurin I., Ramodiharilafy R., Autret G., Clément O., Tharaux P.L., Boilot J.P., Gacoin T., Bouzigues C. (2014). Multifunctional rare-earth vanadate nanoparticles: Luminescent labels, oxidant sensors, and MRI contrast agents. ACS Nano.

[24] Sharma R.K., Mudring A.-V., Ghosh P. (2017). Recent trends in binary and ternary rare-earth fluoride nanophosphors: How structural and physical properties influence optical behavior. J. Lumin..

[25] Feldmann C. (2003). Polyol-mediated synthesis of nanoscale functional materials. Adv. Funct. Mater..

[26] Dang T.M.D., Le T.T.T., Fribourg-Blanc E., Dang M.C. (2012). Influence of surfactant on the preparation of silver nanoparticles by polyol method. Adv. Nat. Sci. Nanosci. Nanotechnol..

[27] Schubert J., Chanana M. (2018). Coating matters: Review on colloidal stability of nanoparticles with biocompatible coatings in biologi-cal media, living cells and organisms. Curr. Med. Chem..

[28] Gao J., Ran X., Shi C., Cheng H., Cheng T., Su Y. (2013). One-step solvothermal synthesis of highly water-soluble, negatively charged superparamagnetic Fe_3_O_4_ colloidal nanocrystal clusters. Nanoscale.

[29] Oleksa V., Macková H., Patsula V., Dydowitzová A., Janoušková O., Horák D. (2020). Doxorubicin-conjugated iron oxide nanoparticles: Surface engineering and biomedical investigation. ChemPlusChem.

[30] Kostiv U., Engstová H., Krajnik B., Šlouf M., Proks V., Podhorodecky A., Ježek P., Horák D. (2020). Monodisperse core-shell NaYF_4_:Yb^3+^/Er^3+^@NaYF_4_:Nd^3+^-PEG-GGGRGDSGGGY-NH_2_ nanoparticles excitable at 808 and 980 nm: Design, surface engineering, and application in life sciences. Front. Chem..

[31] Zasonska B.A., Boiko N., Horák D., Klyuchivska O., Macková H., Beneš M., Babič M., Trchová M., Hromádková J., Stoika R. (2013). The use of hydrophilic poly(*N*,*N*-dimethylacrylamide) for promoting engulfment of magnetic γ-Fe_2_O_3_ nanoparticles by mammalian cells. J. Biomed. Nanotechnol..

[32] Gregori M., Bertani D., Cazzaniga E., Orlando A., Mauri M., Bianchi A., Re F., Sesana S., Minniti S., Francolini M. (2015). Investigation of functionalized poly(*N*,*N*-dimethylacrylamide)-*block*-polystyrene nanoparticles as novel drug delivery system to overcome the blood–brain barrier in vitro. Macromol. Biosci..

[33] Poul L., Ammar S., Jouini N., Fievet F., Villain F. (2003). A synthesis of inorganic compounds (metal, oxide and hydroxide) in polyol medium: A versatile route related to the sol-gel process. J. Sol-Gel. Sci. Tech..

[34] Clayton K.N., Salameh J.W., Wereley S.T., Kinzer-Ursem T.L. (2016). Physical characterization of nanoparticle size and surface mo-dification using particle scattering diffusometry. Biomicrofluidics.

[35] Moore T.L., Rodriguez-Lorenzo L., Hirsch V., Balog S., Urban D., Jud C., Rothen-Rutishauser B., Lattuada M., Petri-Fink A. (2015). Nanoparticle colloidal stability in cell culture media and impact on cellular interactions. Chem. Soc. Rev..

[36] Porfire A., Achim M., Tefas L., Sylvester B., Catala A. (2018). Liposomal nanoformulations as current tumor-targeting approach to cancer therapy. Liposomes.

[37] Andrews K.W., Dyson D.J., Keown S.R. (1967). Interpretation of Electron Diffraction Patterns.

[38] Mishra K., Singh S.K., Singh A.K., Rai M., Gupta B.P., Rai S.B. (2014). New perspective in garnet phosphor: Low temperature synthesis, nanostructures, and observation of multimodal luminescence. Inorg. Chem..

[39] Liang H.J., Chen G.Y., Li L., Liu Y., Qin F., Zhang Z.G. (2009). Upconversion luminescence in Yb^3+^/Tb^3+^-codoped monodisperse NaYF_4_ nanocrystals. Opt. Commun..

[40] Zhang W.J., Chen Q.J., Qian Q., Zhang Q.Y., Jiang Z.H. (2010). Cooperative energy transfer in Tb^3+^/Yb^3+^- and Nd^3+^/Yb^3+^/Tb^3+^-codoped oxyfluoride glasses. Phys. B Condens. Matter..

[41] Debasu M.L., Ananias D., Pinho S.L.C., Geraldes C.F.G.C., Carlos L.D., Rocha J. (2012). (Gd,Yb,Tb)PO_4_ up-conversion nanocrystals for bimodal luminescence–MR imaging. Nanoscale.

[42] Zhang P., He Y., Liu J., Feng J., Sun Z., Lei P., Yuan Q., Zhang H. (2016). Core-shell BaYbF_5_:Tm@BaGdF_5_:Yb,Tm nanocrystals for in vivo trimodal UCL/CT/MR imaging. RSC Adv..

[43] Zheng X., Wang Y., Sun L., Chen N., Li L., Shi S., Malaisamy S., Yan C. (2016). TbF_3_ nanoparticles as dual-mode contrast agents for ultrahigh field magnetic resonance imaging and X-ray computed tomography. Nano Res..

