# Supplementary material for: Colloidally Stable P(DMA-AGME)-Ale-Coated Gd(Tb)F3:Tb3+(Gd3+),Yb3+,Nd3+ Nanoparticles as a Multimodal Contrast Agent for Down- and Upconversion Luminescence, Magnetic Resonance Imaging, and Computed Tomography
Source: Nanomaterials (Basel). 2021 Jan 16;11(1):230. doi: 10.3390/nano11010230 (PMC7830756; doi:10.3390/nano11010230)
Supplement: Supplementary file 1 [file nanomaterials-11-00230-s001.pdf]

Supporting Information

Article

Colloidally Stable P(DMA-AGME)-Ale-Coated Gd(Tb)F$_3$:Tb^{3+}(Gd^{3+}),Yb^{3+},Nd^{3+} Nanoparticles as a Multimodal Contrast Agent for Down- and Up-conversion Luminescence, Magnetic Resonance Imaging, and Computed Tomography

Oleksandr Shapoval [1,*], Viktoriia Oleksa [1], Miroslav Šlouf [1], Volodymyr Lobaz [1], Olga Trhlíková [1], Marcela Filipová [1], Olga Janoušková [1], Hana Engstová [2], Jan Pankrác [3], Adam Modrý [3], Vít Herynek [3], Petr Ježek [2], Luděk Šefc [3] and Daniel Horák [1,*]

[1] Institute of Macromolecular Chemistry, Czech Academy of Sciences, 162 06 Prague 6, Czech Republic; oleksa@imc.cas.cz (V.O.); slouf@imc.cas.cz (M.S.); lobaz@imc.cas.cz (V.L.); trhlikova@imc.cas.cz (O.T.); filipova@imc.cas.cz (M.F.); janouskova324@gmail.com (O.J.)
[2] Institute of Physiology, Czech Academy of Sciences, 142 20 Praha 4, Czech Republic; hana.engstova@fgu.cas.cz (H.E.); Petr.Jezek@fgu.cas.cz (P.J.)
[3] Center for Advanced Preclinical Imaging, First Faculty of Medicine, Charles University, 120 00 Prague 2, Czech Republic; jan.pankrac@lf1.cuni.cz (J.P.); adam.modry@lf1.cuni.cz (A.M.); vit.herynek@lf1.cuni.cz (V.H.); sefc@cesnet.cz (L.S.)
* Correspondence: shapoval@imc.cas.cz (O.S.); horak@imc.cas.cz (D.H.); Tel.: +420-296-809-260 (D.H.)

Table S1. Characterization of GdF$_3$@PDMAnanoparticles.

**Shell**	M_w (g/mol)	**Polymer/particles** (w/w)	**Colloidal stability in 0.01M PBS**		
			**τ** (days)	D_h (nm)	*PD*
PDMA$_{8k}$-Ale	**8,000**	**1/2**	*	*	*
		**1/1**	**7**	**105**	**0.2**
PDMA$_{11k}$-Ale	**11,000**	**1/2**	**2**	**205**	**0.4**
		**1/1**	**7**	**73**	**0.2**
PDMA$_{39k}$-Ale	**39,000**	**1/2**	**1**	**1358**	**0.4**
		**1/1**	**2**	**767**	**0.2**

* - Unstable dispersion; τ – time; D_h - hydrodynamic diameter (DLS); *PD* – polydispersity (DLS).

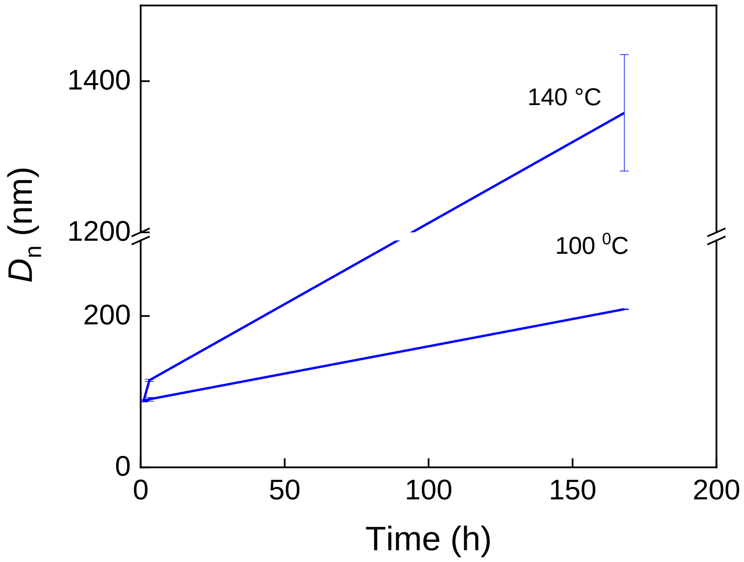

Figure S1. Dependence of hydrodynamic diameter D_h of GdF_3:Tb^{3+},Yb^{3+},Nd^{3+}@P(DMA-AGME)-Ale nanoparticles in water on time of storage. The particles were synthesized at 100 or 140 °C.

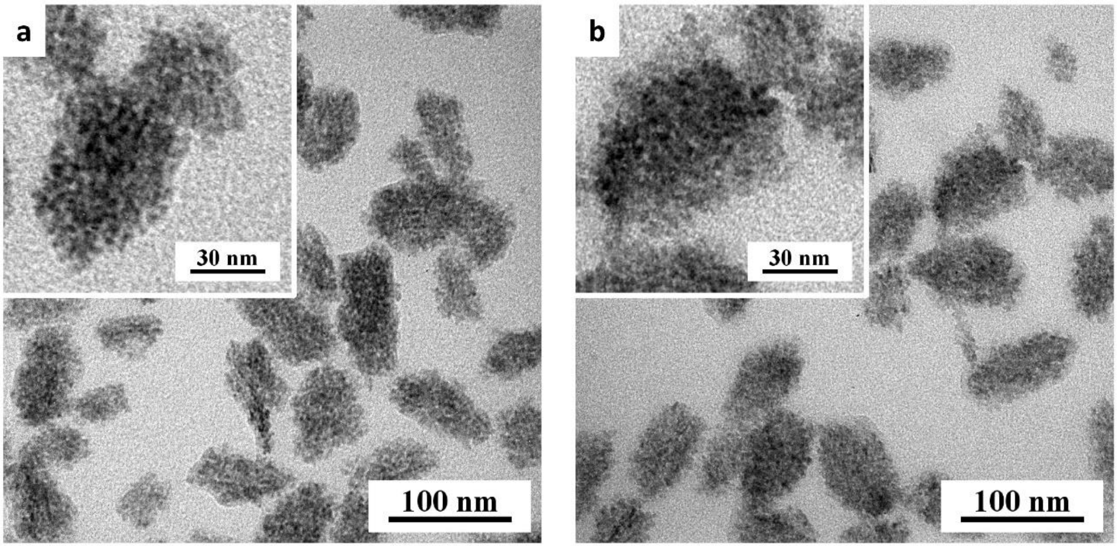

Figure S2. High magnification TEM micrographs of P(DMA-AGME)-Ale-coated (**a**) GdF_3:Tb^{3+},Yb^{3+},Nd^{3+} and (**b**) TbF_3:Gd^{3+},Yb^{3+},Nd^{3+} nanoparticles. The insets display the very small individual particles with sizes <~5 nm.

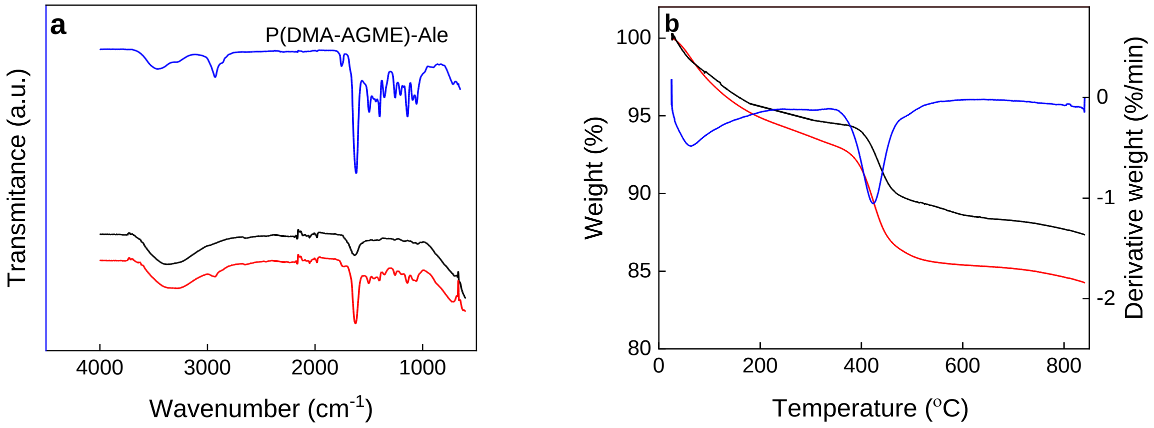

Figure S3. (**a**) FTIR spectra and (**b**) TGA and DTGA (differential thermogravimetric analysis) of P(DMA-AGME)-Ale-coated $GdF_3:Tb^{3+},Yb^{3+},Nd^{3+}$ (black) and $TbF_3:Gd^{3+},Yb^{3+},Nd^{3+}$ nanoparticles (red).

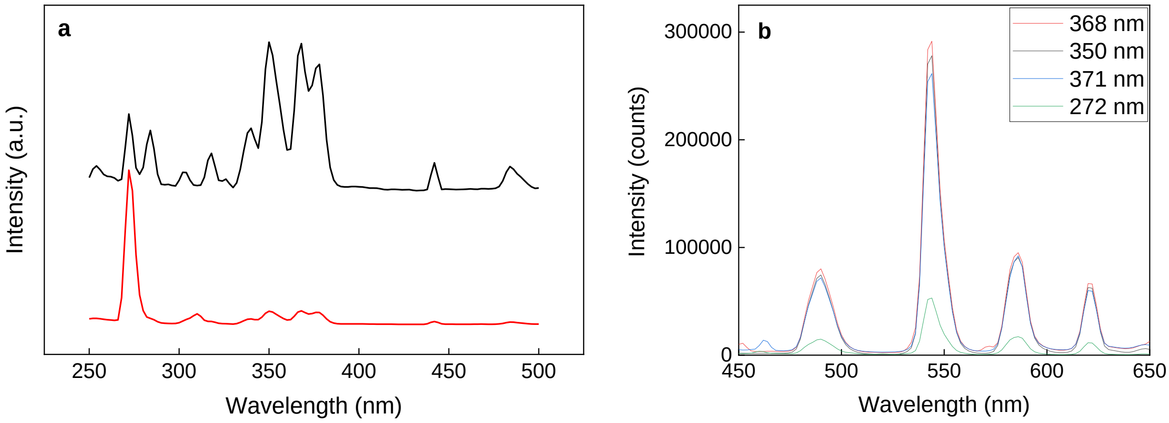

Figure S4. (**a**) DC excitation spectra of P(DMA-AGME)-Ale-coated $GdF_3:Tb^{3+},Yb^{3+},Nd^{3+}$ (red) and $TbF_3:Gd^{3+},Yb^{3+},Nd^{3+}$ nanoparticles (black); (**b**) DC emission spectra $TbF_3:Gd^{3+},Yb^{3+},Nd^{3+}$@P(DMA-AGME)-Ale nanoparticles excited at different wavelengths.

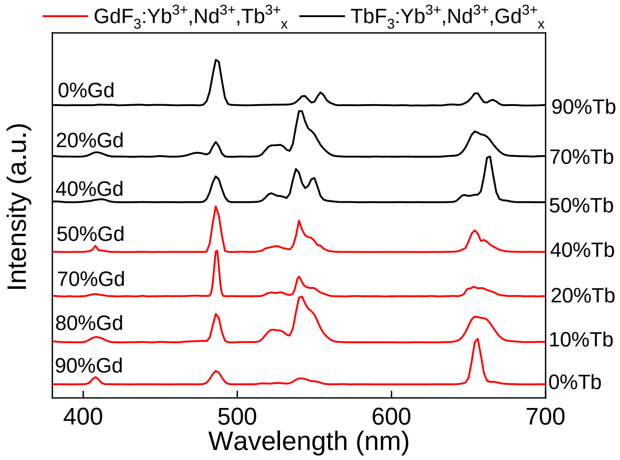

Figure S5. UC photoluminescence emission spectra of P(DMA-AGME)-Ale-coated GdF_3:Tb^{3+},Yb^{3+},Nd^{3+} and TbF_3:Gd^{3+},Yb^{3+},Nd^{3+} nanoparticles doped with different concentrations of Gd^{3+} and Tb^{3+} ions; 980 nm excitation, particle concentration 1 mg/ml, and power density 5 W/cm^2.

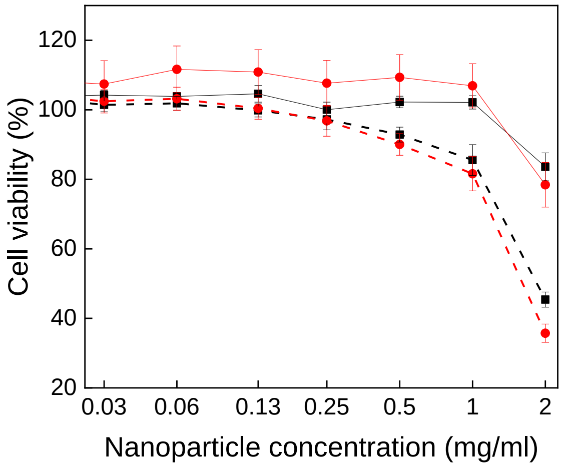

Figure S6. Cytotoxicity of P(DMA-AGME)-Ale-coated GdF_3:Tb^{3+},Yb^{3+},Nd^{3+} (black) and TbF_3:Gd^{3+},Yb^{3+},Nd^{3+} nanoparticles (red) incubated with Hela (dashed line) and HF cells (solid line) for 72 h.

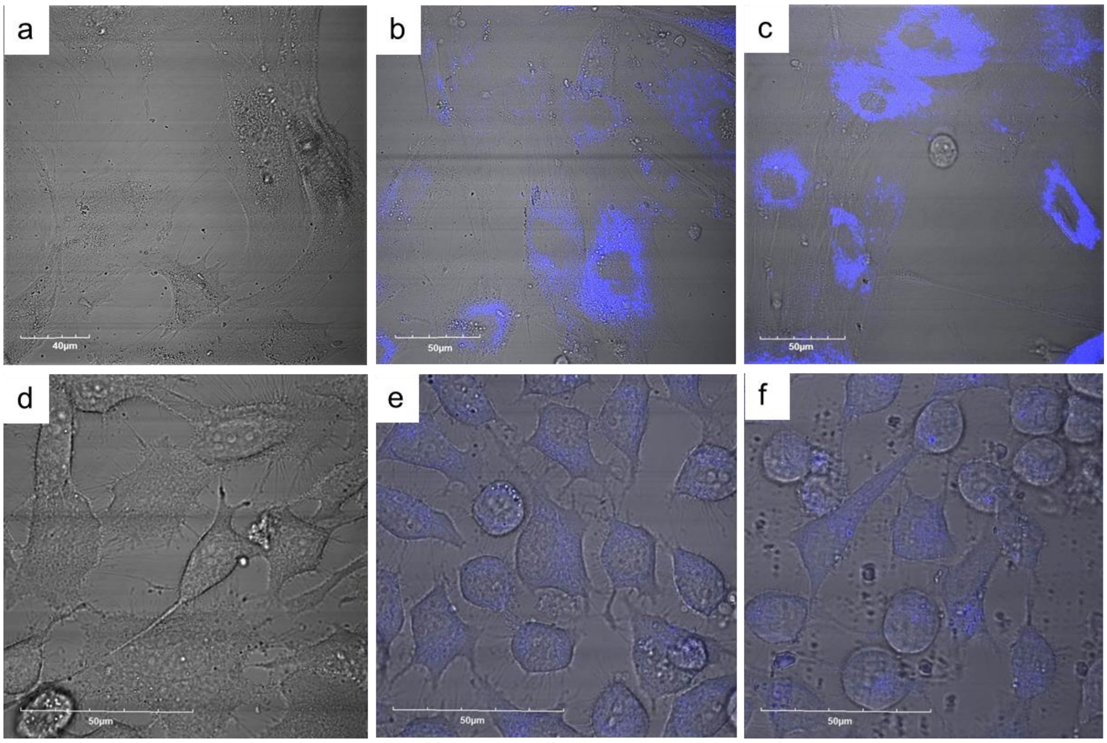

Figure S7. Laser scanning confocal micrographs of (a-c) HF and (d-f) HeLa cells (a, d) before (negative control) and after treatment with (b, e) TbF_3:Gd^{3+},Yb^{3+},Nd^{3+}@P(DMA-AGME)-Ale and (c, f) GdF_3:Tb^{3+},Yb^{3+},Nd^{3+}@P(DMA-AGME)-Ale nanoparticles.

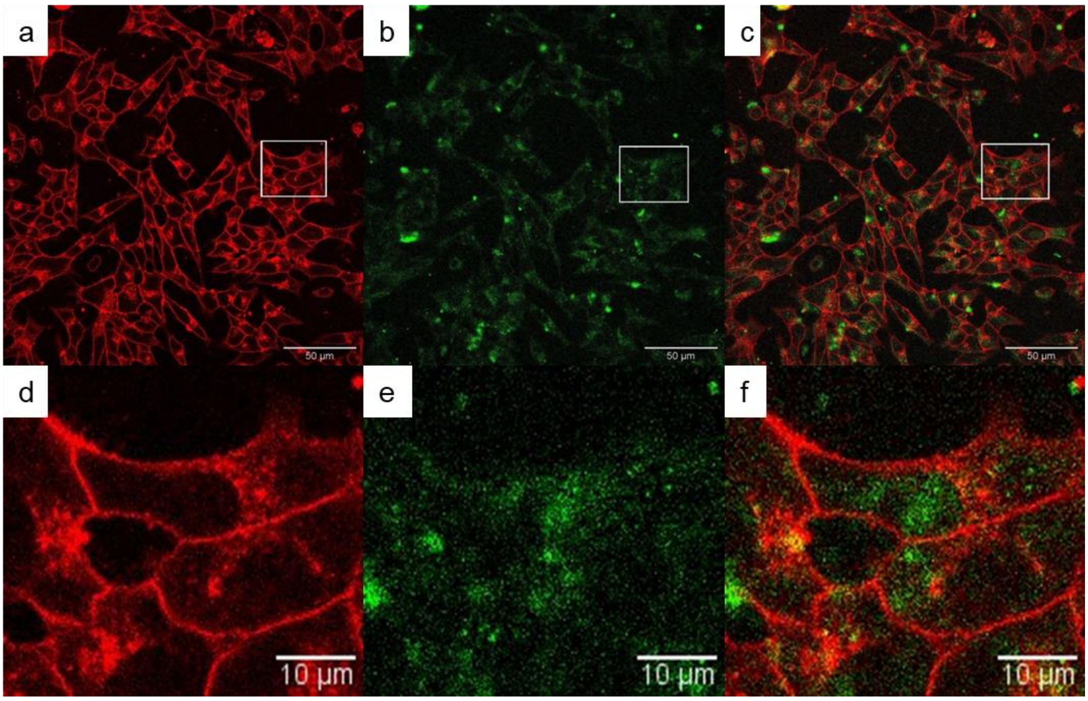

Figure S8. (**a-f**) Confocal micrographs of biodistribution of TbF$_3$:Gd^{3+},Yb^{3+},Nd^{3+}@P(DMA-AGME)-Ale nanoparticles in INS cells at 808 nm excitation with a laser power of 30-50 mW. (**d-f**) Detailed micrographs of (**a-c**). (**a, d**) CellMask™ deep red-stained cell membrane, (**b, e**) nanoparticles (green), and (**c, f**) overlay of (**a, b**) and (**d, e**), respectively.

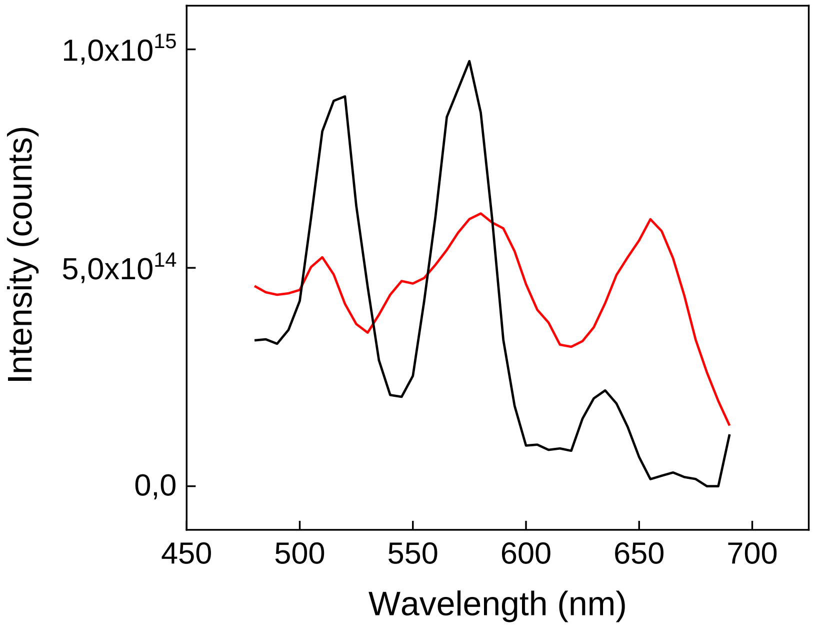

Figure S9. Emission spectra of pure GdF$_3$:Tb^{3+},Yb^{3+},Nd^{3+}@P(DMA-AGME)-Ale nanoparticles (black) and nanoparticles localized in the HepG2 cells (red).

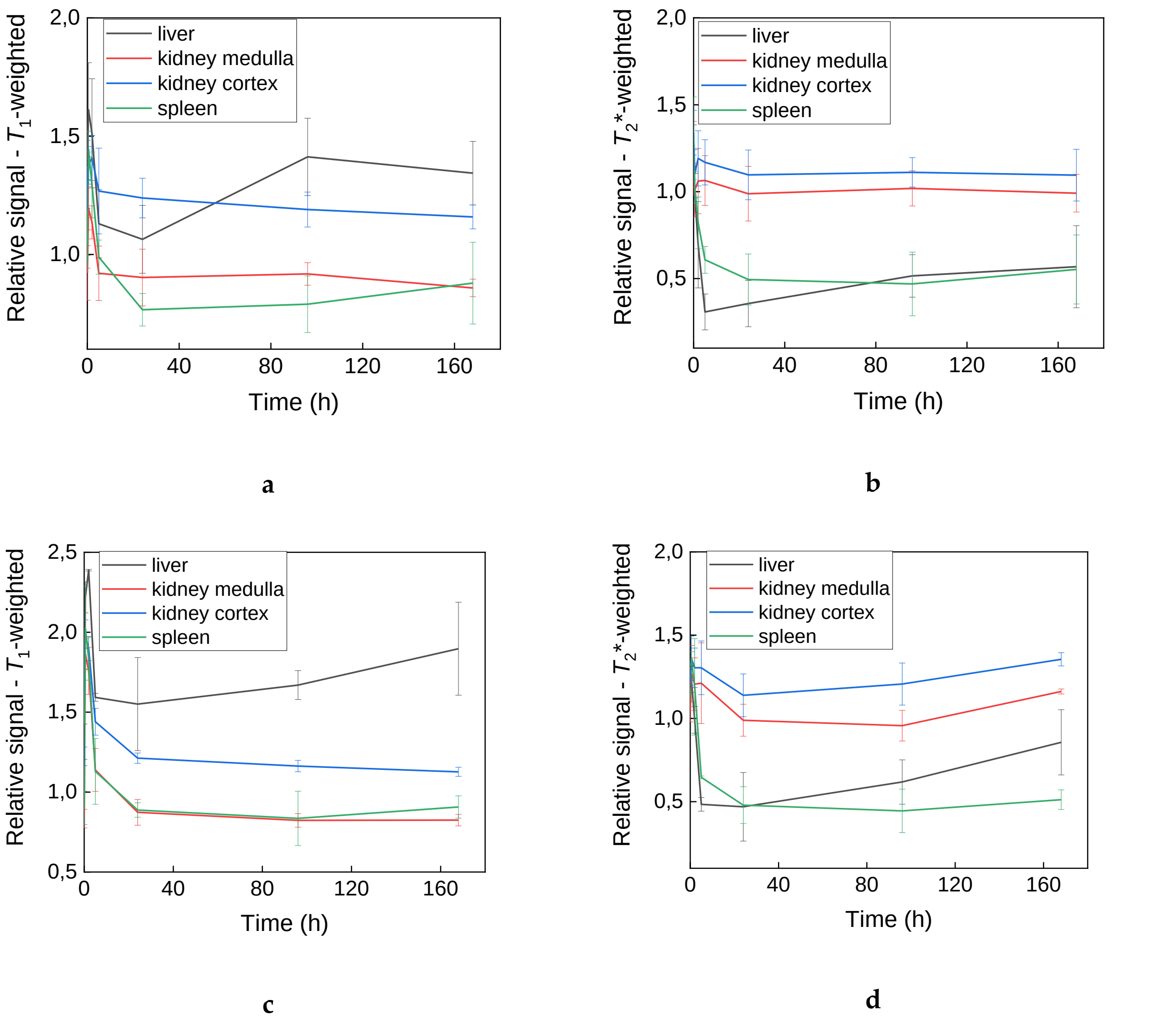

Figure S10. (**a, c**) Relative T_1 and (**b, d**) T_2* signal evolution in the liver, kidney (medulla and cortex), and spleen after administration of P(DMA-AGME)-Ale-coated (**a, b**) $TbF_3:Gd^{3+},Yb^{3+},Nd^{3+}$ and (**c, d**) $GdF_3:Tb^{3+},Yb^{3+},Nd^{3+}$ nanoparticles in mice.

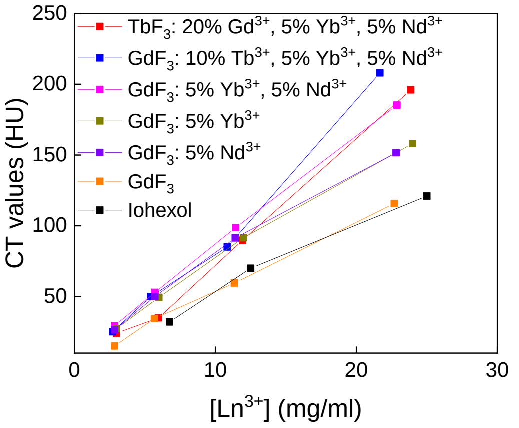

Figure S11. Dependence of CT value of $Gd(Tb)F_3:Tb^{3+}(Gd^{3+}),Yb^{3+},Nd^{3+}$@P(DMA-AGME)-Ale nanoparticles and Iohexol on the concentration. HU - Hounsfield units.

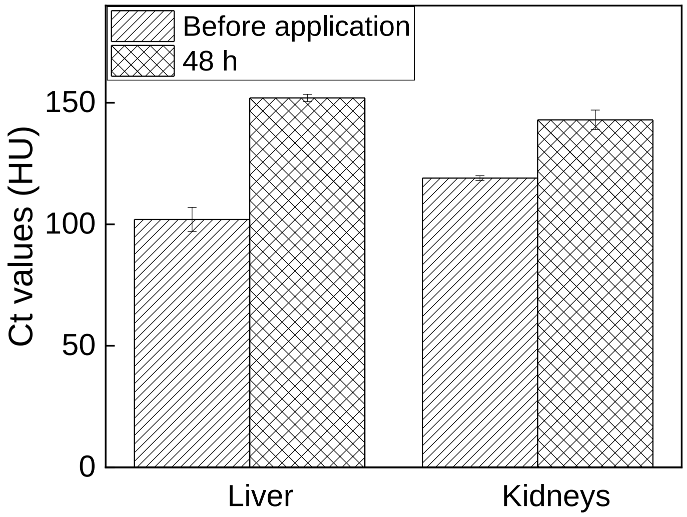

Figure S12. Relative signal evolution of the CT contrast (HU) in the liver and kidneys calculated from pre-injection to 48 h post-injection of GdF_3:Tb^{3+},Yb^{3+},Nd^{3+}@P(DMA-AGME)-Ale nanoparticles. Error bars represent the standard deviation from two independent images.

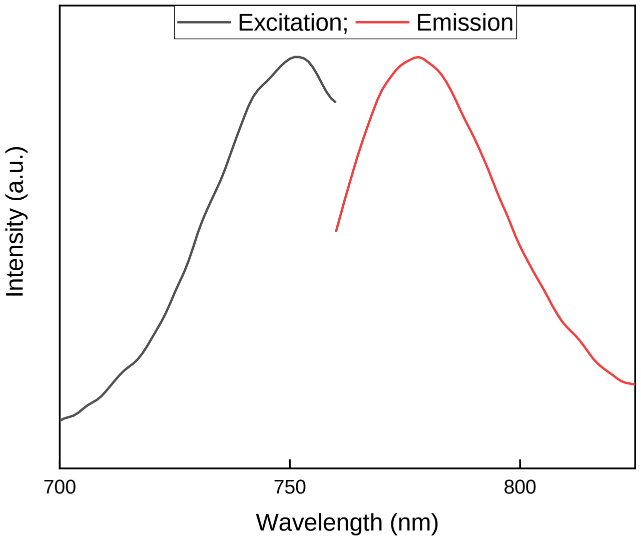

Figure S13. Photoluminescence spectra of Cy7-modified Gd(Tb)F_3:Tb^{3+}(Gd^{3+}),Yb^{3+},Nd^{3+}@P(DMA-AGME)-Ale nanoparticles.

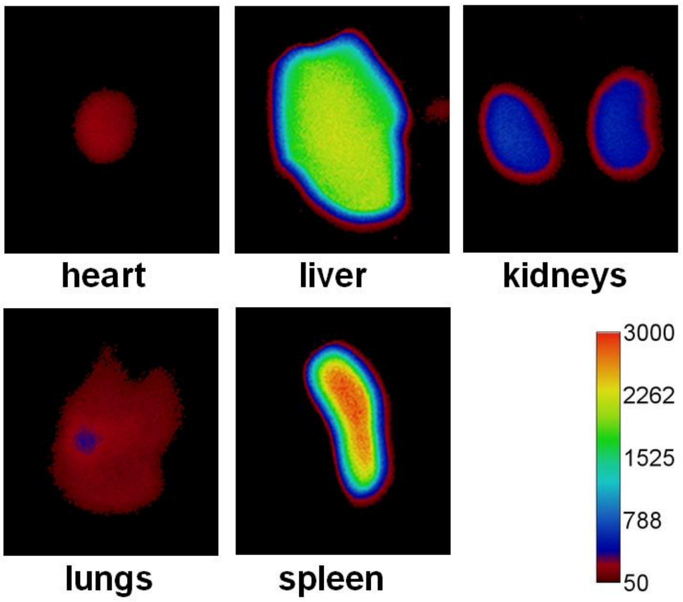

Figure S14. Fluorescence imaging of the excised mouse organs 168 h after administration of GdF_3:Tb^{3+},Yb^{3+},Nd^{3+}@P(DMA-AGME)-Ale-Cy7 nanoparticles.
